# Supplementary material for: De novo design of protein interactions with learned surface fingerprints
Source: Nature. 2023 Apr 26;617(7959):176–84. doi: 10.1038/s41586-023-05993-x (PMC10131520; doi:10.1038/s41586-023-05993-x)
Supplement: Supplementary file 1 — Supplementary Figs. 1–23 and Supplementary Tables 1–7. [file 41586_2023_5993_MOESM1_ESM.pdf]

nature portfolio

https://doi.org/10.1038/s41586-023-05993-x

Supplementary information

De novo design of protein interactions with learned surface fingerprints

In the format provided by the
authors and unedited

Supplemental Material

De novo design of protein interactions with learned surface fingerprints

Pablo Gainza*[1,†], Sarah Wehrle*[1], Alexandra Van Hall-Beauvais*[1], Anthony Marchand*[1], Andreas Scheck*[1], Zander Harteveld[#1], Stephen Buckley[#1], Dongchun Ni[#2], Shuguang Tan[#3], Freyr Sverrisson[1], Casper Goverde[1], Priscilla Turelli[4], Charlène Raclot[4], Alexandra Teslenko[5], Martin Pacesa[1], Stéphane Rosset[1], Sandrine Georgeon[1], Jane Marsden[1], Aaron Petruzzella[6], Kefang Liu[3], Martin Pacesa[1], Zepeng Xu[3], Yan Chai[3], Pu Han[3], George F. Gao[3], Elisa Oricchio[6], Beat Fierz[5], Didier Trono[4], Henning Stahlberg[2], Michael Bronstein[7,◊], Bruno E. Correia[1,◊]

1. Laboratory of Protein Design and Immunoengineering, School of Life Sciences, École Polytechnique Fédérale de Lausanne, and Swiss Institute of Bioinformatics, Lausanne, Switzerland
2. Laboratory of Biological Electron Microscopy, Institute of Physics, School of Basic Science, École Polytechnique Fédérale de Lausanne, and Dep. of Fund. Microbiology, Faculty of Biology and Medicine, University of Lausanne, Lausanne, Switzerland
3. CAS Key Laboratory of Pathogen Microbiology and Immunology, Institute of Microbiology, Chinese Academy of Sciences, Beijing, China
4. Laboratory of Virology and Genetics, School of Life Sciences, École Polytechnique Fédérale de Lausanne, Lausanne, Switzerland
5. Laboratory of Biophysical Chemistry of Macromolecules, School of Basic Sciences, Institute of chemical sciences and engineering (ISIC), École Polytechnique Fédérale de Lausanne, Lausanne, Switzerland
6. Swiss Institute for Experimental Cancer Research, School of Life Sciences, École Polytechnique Fédérale de Lausanne, Lausanne, Switzerland
7. Department of Computer Science, University of Oxford, Oxford, UK

† Present address: Monte Rosa Therapeutics AG, Basel, Switzerland

* equal contribution; # equal contribution; ◊ corresponding authors

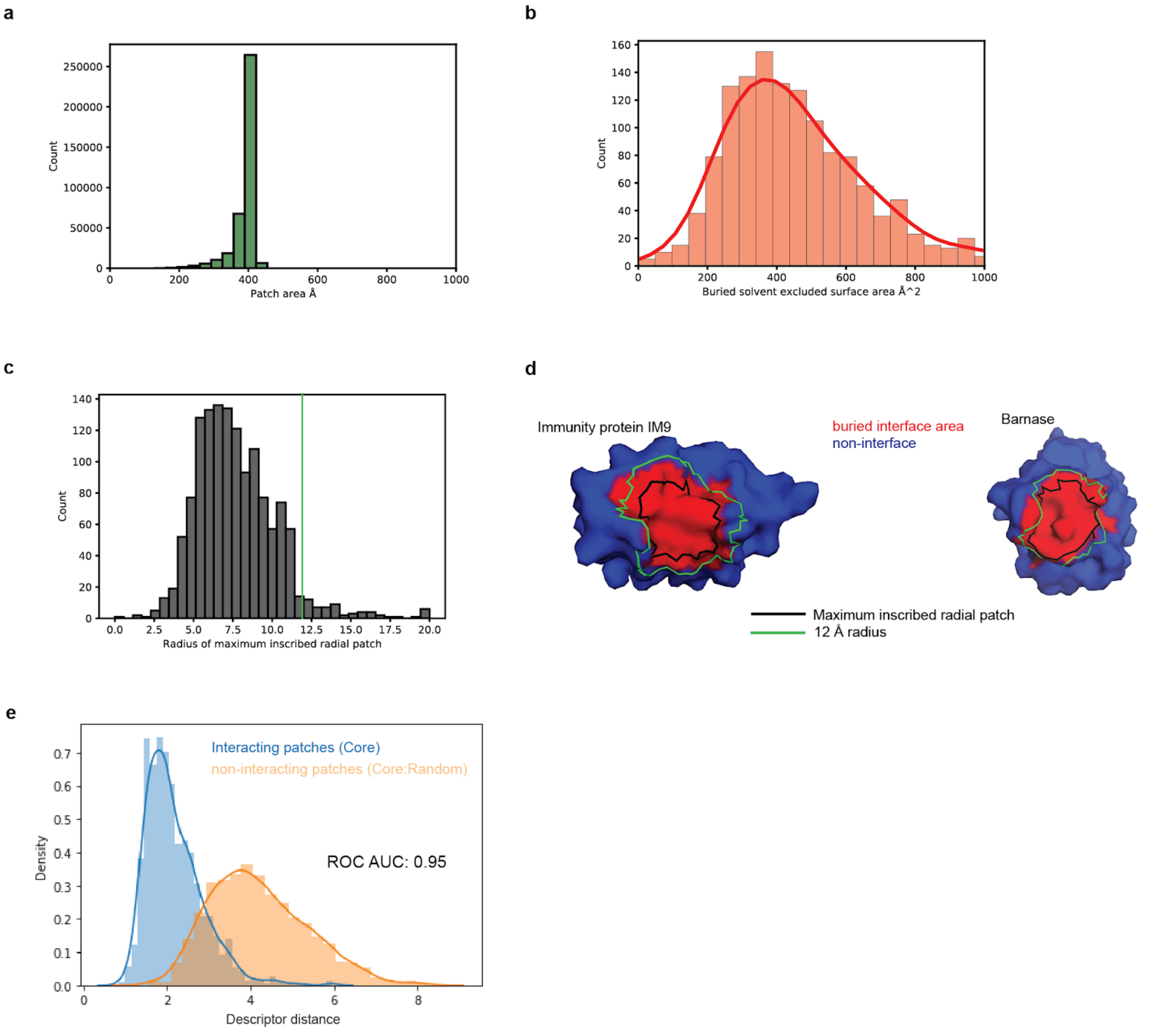

Supplementary Figure S1: Modeling buried surfaces as radial patches. a, Histogram of the patch areas of thousands of randomly selected protein patches with a fixed radius of 12 Å. **b,** Histogram of the area of the buried surface area on 1380 dimeric PPIs. We note that areas are computed for only one of the proteins (i.e. each subunit in a PPI is computed separately), and that we used the solvent excluded surface area, while other authors report buried areas on the solvent accessible area that include the buried surface area of both proteins (see methods). **c,** Size of the maximum inscribed radial patch for the 1380 proteins (see methods). Patch area for the radius used here (12 Å), using a set of 30,000 randomly selected patches. **d,** Example of the buried interface area for two well known, high affinity binders, Immunity Protein IM9 (PDB ID: 1EMV) and the protein Barnase (PDB ID: 1BRS). The buried interface of each protein when bound to its partner is shown in red. The maximum inscribed radial patch's circumference is shown in black, and the circumference of a patch with radius 12 Å is shown in green. **e,** Histogram of similarities between MaSIF-search fingerprint similarity between: (blue) pairs of patches that are co-crystallized from transient PPIs, with the fingerprint computed for the patch centered on the largest inscribed radial patch, and (orange) pairs of patches where one was taken from the center of the interface of a random PPI and the other was taken from a random patch surface.

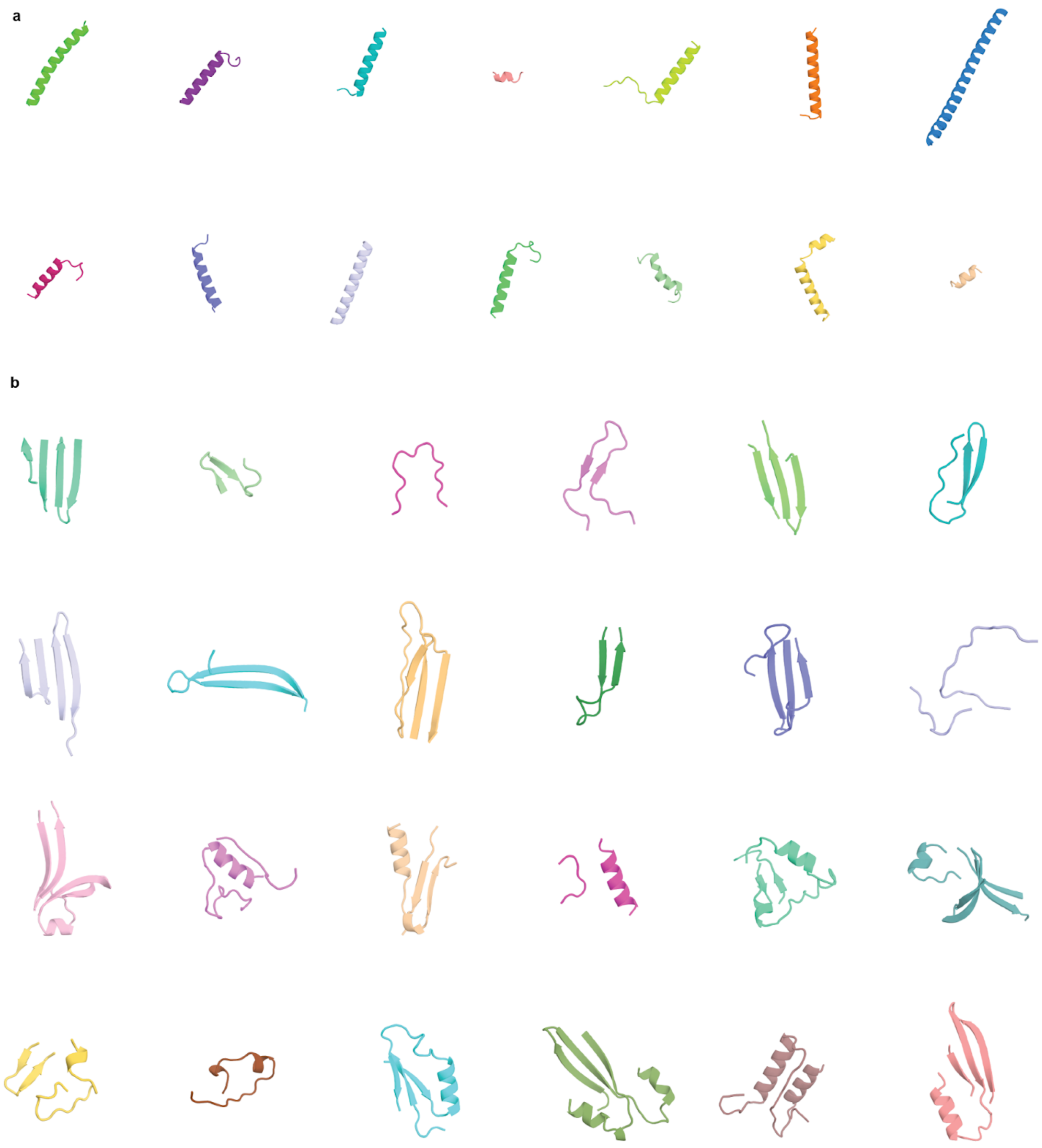

Supplementary Figure S2: Overview of helical and non-helical seeds used in the recovery benchmark. Examples of **a,** helical seed, **b,** non-helical seeds that were extracted for the recovery benchmark.

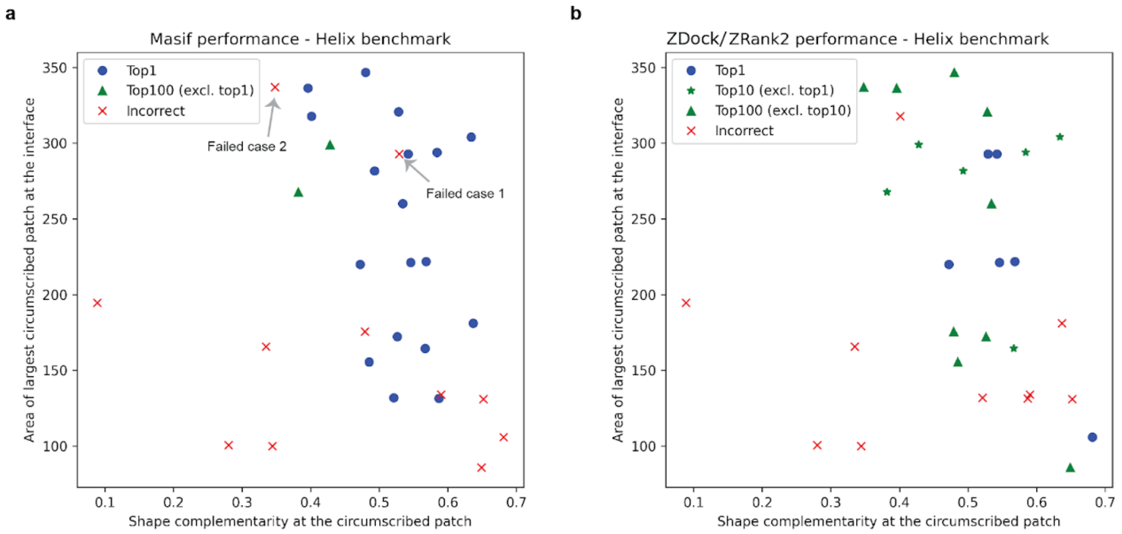

c

	Benchmark cases solved by MaSIF-seed but not by ZDock/ZRank2	Benchmark cases solved by ZDock/ZRank2 but not by MaSIF-seed	Benchmark cases solved by both MaSIF-seed and ZDock/ZRank2
In the Top 1	14	2	4
In the Top 10	10	4	8
In the Top 100	4	5	16

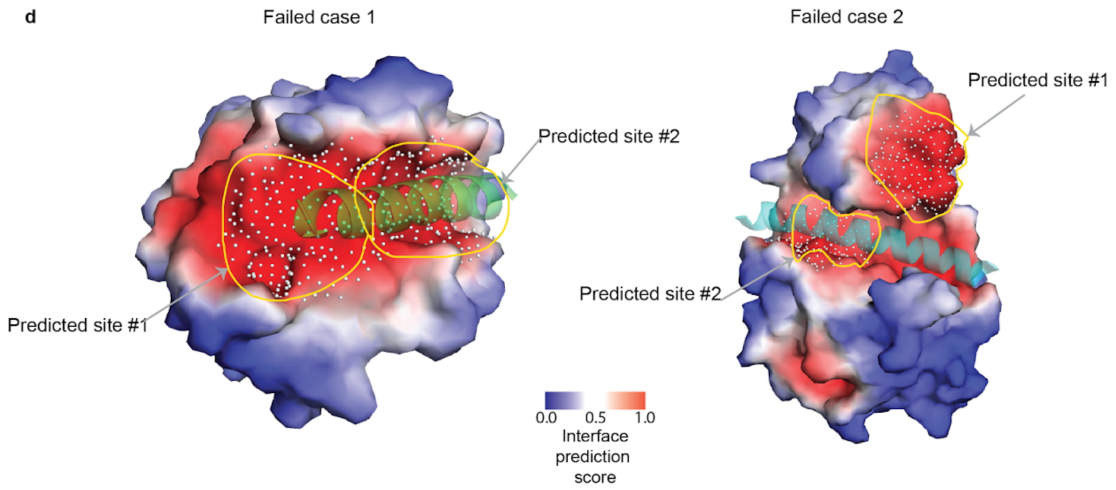

Supplementary Figure S3: Analysis of successful/failed helical benchmark cases and comparison between MaSIF-seed and ZDock/ZRank2 performance. a-b, Plotting of Top 1, Top 10, Top 100 and failed cases for MaSIF-seed and ZDock/ZRank2, showing the maximum circumscribed patch area in the buried interface (y-axis) and the median shape complementarity for vertices of that patch (x-axis) for **a,** MaSIF-seed, and **b**, ZDock/ZRank2. **c**, Comparison of cases solved by only MaSIF-seed, only ZDock/ZRank2, or both MaSIF-seed and ZDock/ZRank2 in the Top 1, top 10 or Top 100 rank. **d**, Analysis of two cases that showed both a large circumscribed patch and high complementarity at that patch where MaSIF-seed failed. Left (Failed case 1) shows the BHRF1:Bak BH3 complex (PDB ID: 2XPX); right (Failed case 2) shows proteinase A complexed with a IA3 mutant (PDB ID: 1G0V). In both cases, MaSIF-seed failed because it identified a different site as the top site, but increasing the number of sites explored to the top two resulted in successful predictions. The white dots on the surface denote the predicted site patches.

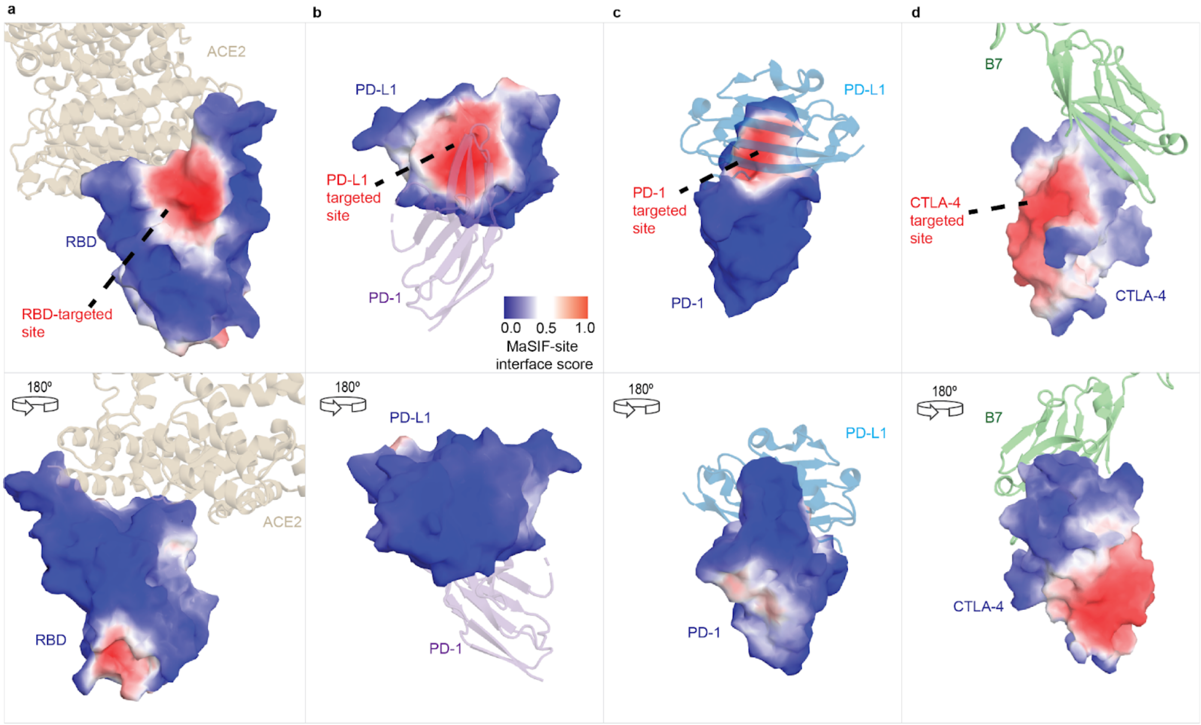

Supplementary Figure S4: MaSIF-site target site prediction on SARS-CoV-2 RBD, PD-L1, PD-1, and CTLA-4. Surface mode shows a MaSIF-site per-surface-vertex regression score on the propensity of each point on the surface to form an interface ranging from 0 (blue) to 1 (red) **a-c**, Predictions on each target, with the natural ligand of the target shown in cartoon representation as a reference. The structures highlight the predicted site and the bottom row shows a 180 degree rotation. **a**, MaSIF-site prediction on SARS-CoV-2 RBD (PDB ID: 6M17), with the RBD shown in surface and the ACE2 in beige. **b**, Prediction on PD-L1 (PDB ID: 5JDS), with PD-1 shown in purple. **c**, Prediction on PD-1 (PDB ID: 4ZQK) with the natural binder PD-L1 shown in cyan. **d**, Prediction on CTLA-4 (PDB ID: 5GGV) with the natural binding partner B7 (PDB ID: 1I8L) shown in light green.

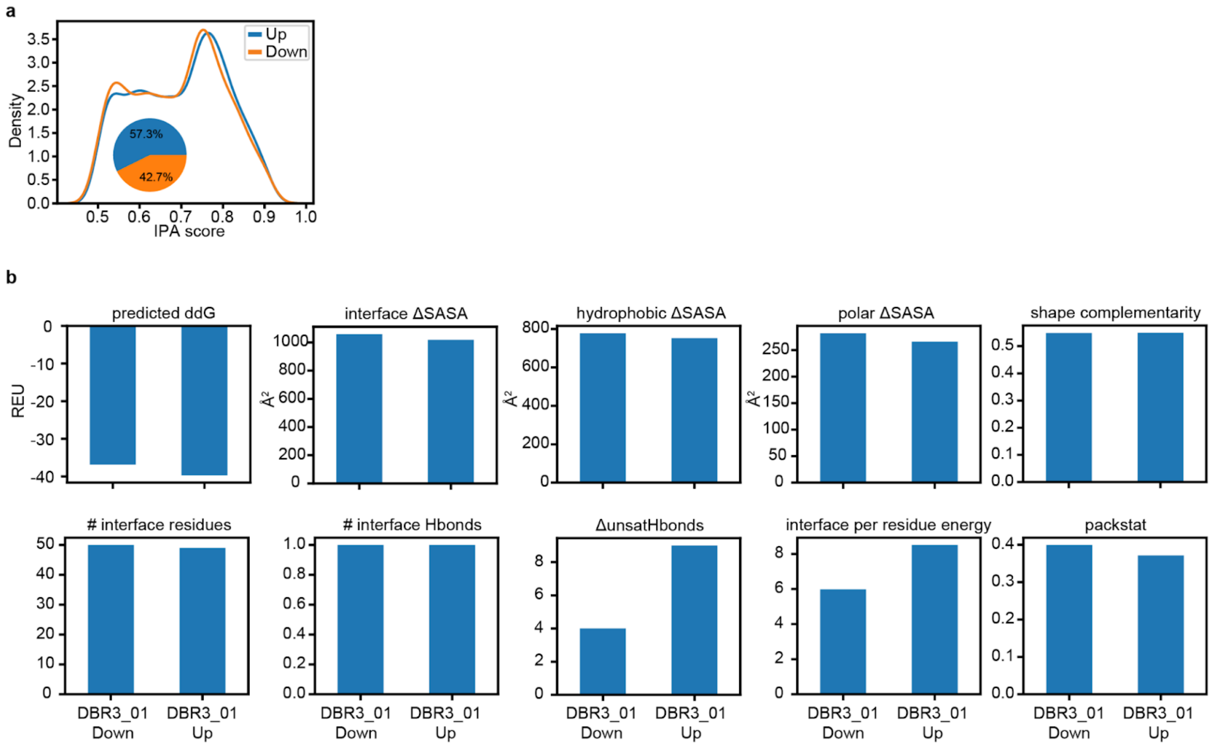

Supplementary Figure S5: RBD-binder metrics for up- and down-orientations . a, Distribution of the IPA scores for the seeds of the up- and down-orientations and respective cluster sizes. **b**, Interface metrics (n=1) of the DBR3_01 model in complex with ACE2 for the up- and down-orientations were computed using Rosetta's interface analyzer. The following Rosetta metrics are shown: predicted ddG = change in Rosetta energy of separated versus complexed binding partners, interface ΔSASA = solvent accessible surface area buried at the interface, hydrophobic ΔSASA = solvent accessible surface area buried at the interface that is hydrophobic, polar ΔSASA = solvent accessible surface area buried at the interface that is polar, shape complementarity = Lawrence and Coleman shape complementarity of the interface surfaces, # interface residues = number of residues at the interface, # interface Hbonds = number of hydrogen bonds across the interface, ΔunsatHbonds = number of buried, unsatisfied hydrogen bonds at the interface, interface per residue energy = average Rosetta energy of each interface residue, packstat = Rosetta's packing statistic score for the interface ranging from 0 (low packing) to 1 (high packing).

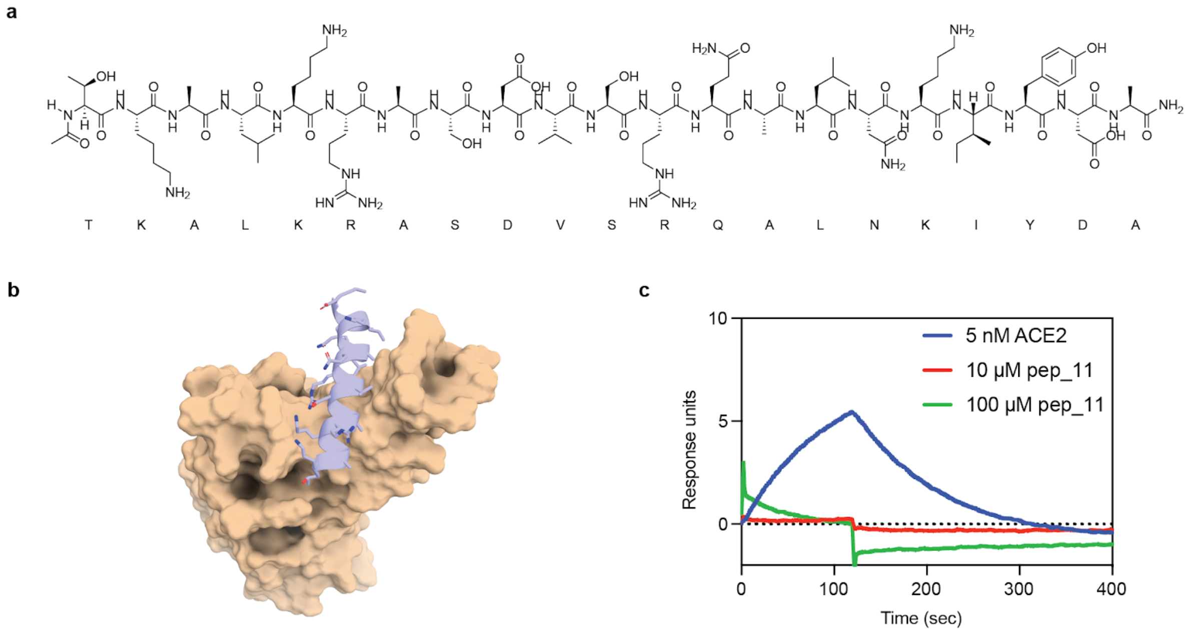

Supplementary Figure S6: Binding seed identified by MaSIF tested as a synthetic peptide. a, Structure of the synthesized binding seed. **b**, MaSIF prediction of seed (lavender) binding to RBD (wheat). **c**, SPR data of high concentration of the peptide flowing over RBD. No binding signal is observed for the peptide.

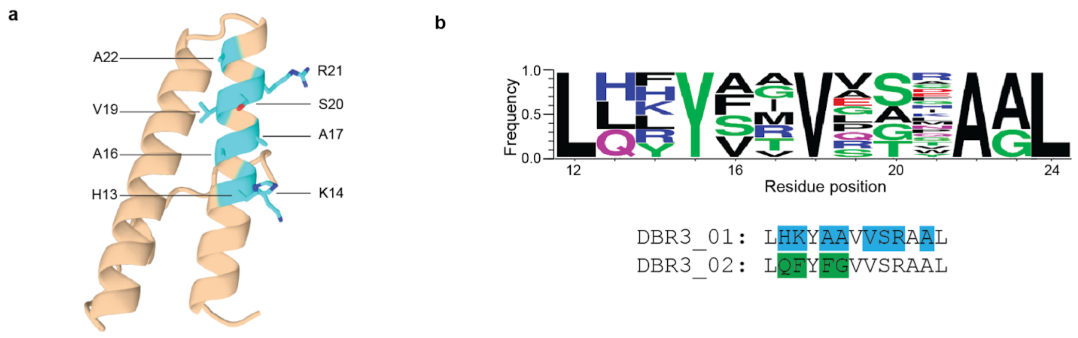

Supplementary Figure S7: Directed Library for DBR3_01. **a**, Position of residues included in a combinatorial library to improve binding affinity. **b**, Sequence logo plot of specific mutations allowed within the library. The sequences list the residues mutated in DBR3_01 (highlighted in blue) and the mutations gained through the library in DBR3_02 (green).

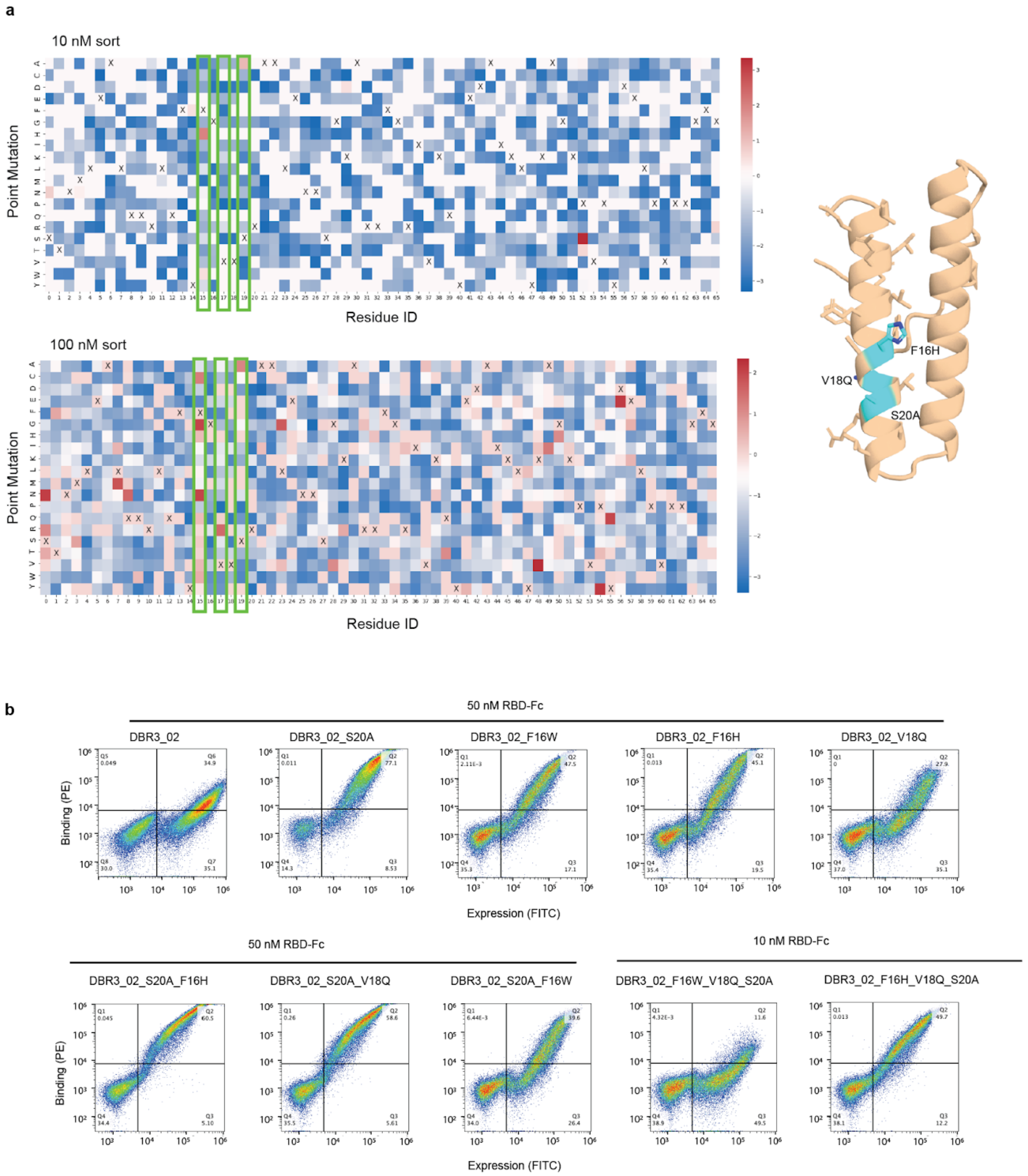

Supplementary Figure S8: SSM of DBR3_02. a, Heat maps of DBR3_02 SSM at two concentrations of RBD-Fc. X indicates the original amino acid of DBR3_02. Red indicates an enrichment of the mutation in the binding population, blue indicates an enrichment in the non-binding population. Three positions, green box, were enriched in both concentrations. The positions of these mutations are highlighted on the DBR3_03 structure. **b**, Yeast display of DBR3_02 with mutations from the SSM introduced shows increase in affinity to RBD.

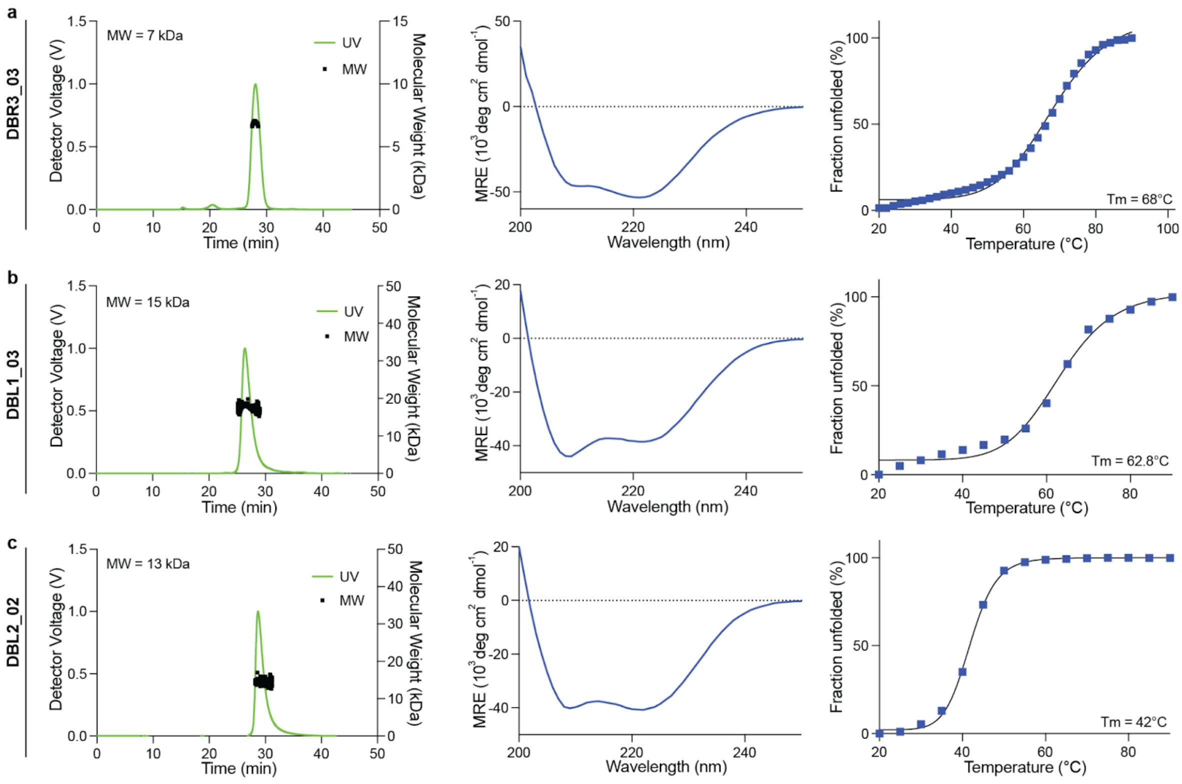

Supplementary Figure S9: Biophysical characterization of the designed binders. From left to right: The oligomeric status was determined via multi-angled light scattering (MALS). Folding was measured using circular dichroism. Thermal stability was determined by plotting the ellipticity at 218 nm at increasing temperatures. **a**, DBR3_03, **b**, DBL1_03, **c**, DBL2_02.

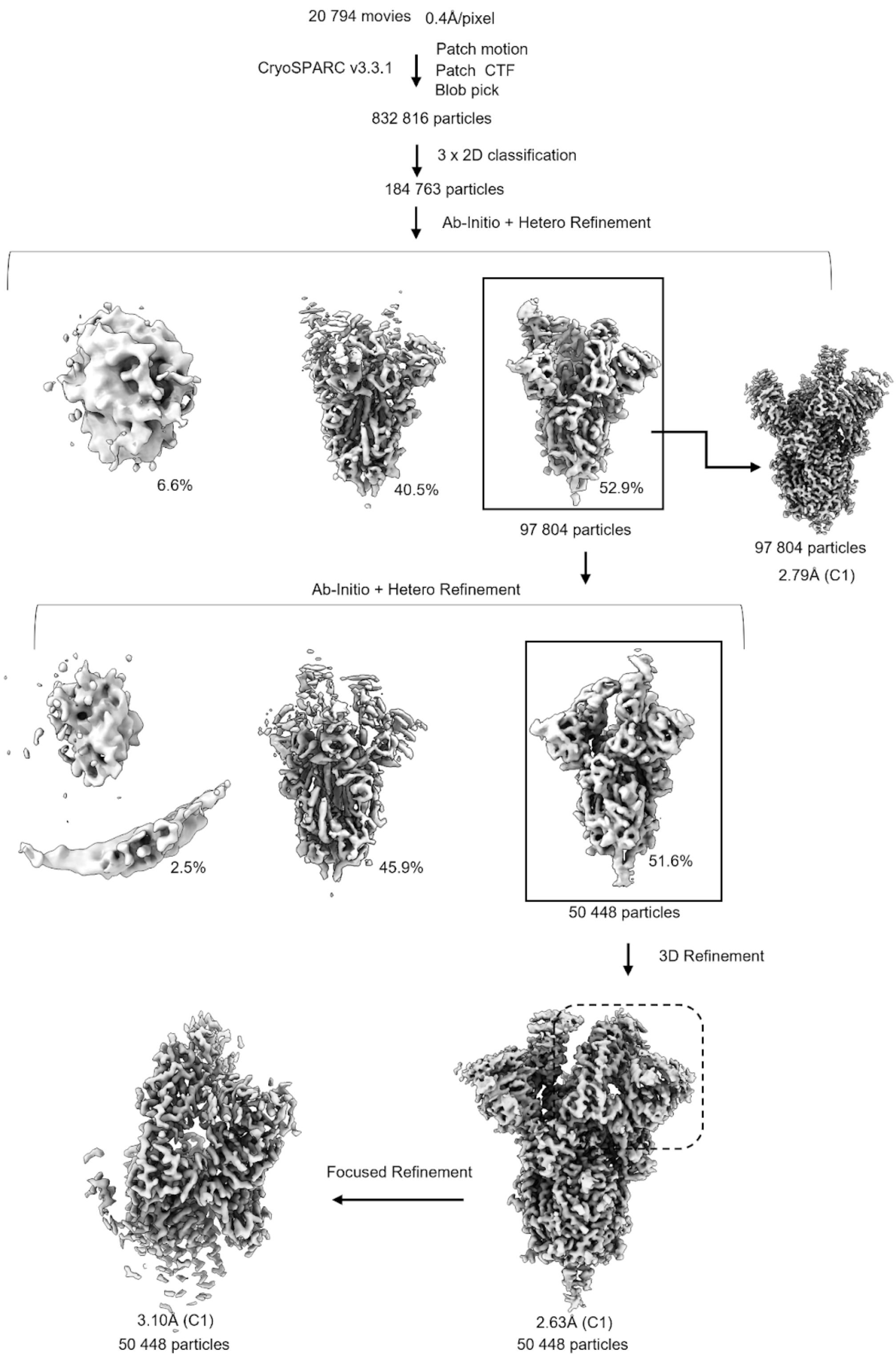

Supplementary Figure S10: Cryo-EM data processing of the D614G Spike-DBR3_03 complex. Image processing workflows performed in CryoSPARC v.3.3.1.

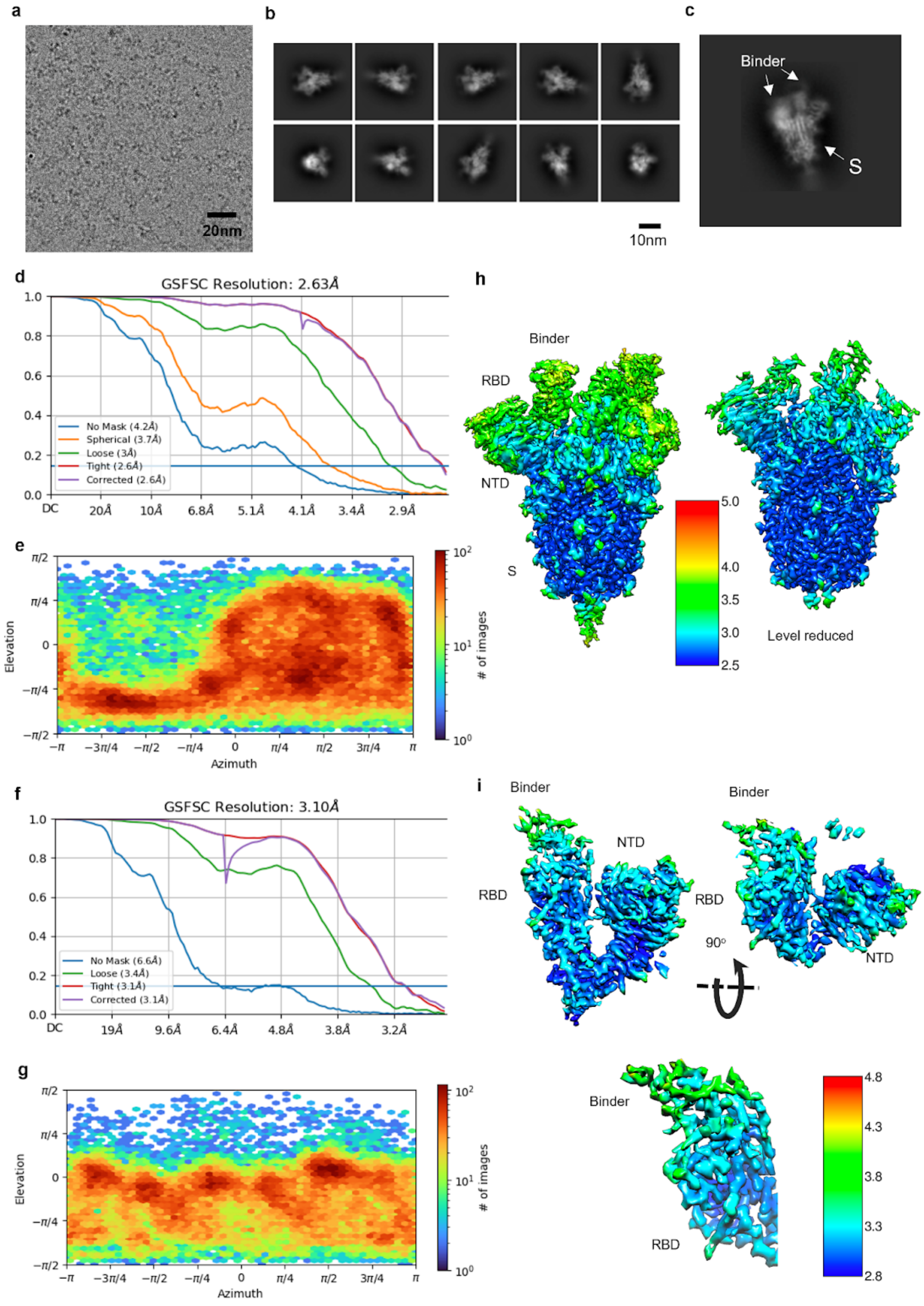

Supplementary Figure S11: Details of Cryo-EM data processing for D614G Spike-DBR3_03 complex. **a,** A representative raw micrograph of the Cryo-EM sample for D614G Spike-binder complex. 20,794 micrographs of such similar quality were acquired for this complex. **b,** The 2D classes of the D614G Spike-binder complex. **c,** A representative 2D class. **d,** Direction distribution of the particle alignment and **e,** FSC curves of the final overall map. **f,** Direction distribution and **g,** FSC curves of the locally refined map. **h,i,** Local resolution distribution of the overall and focused refined maps.

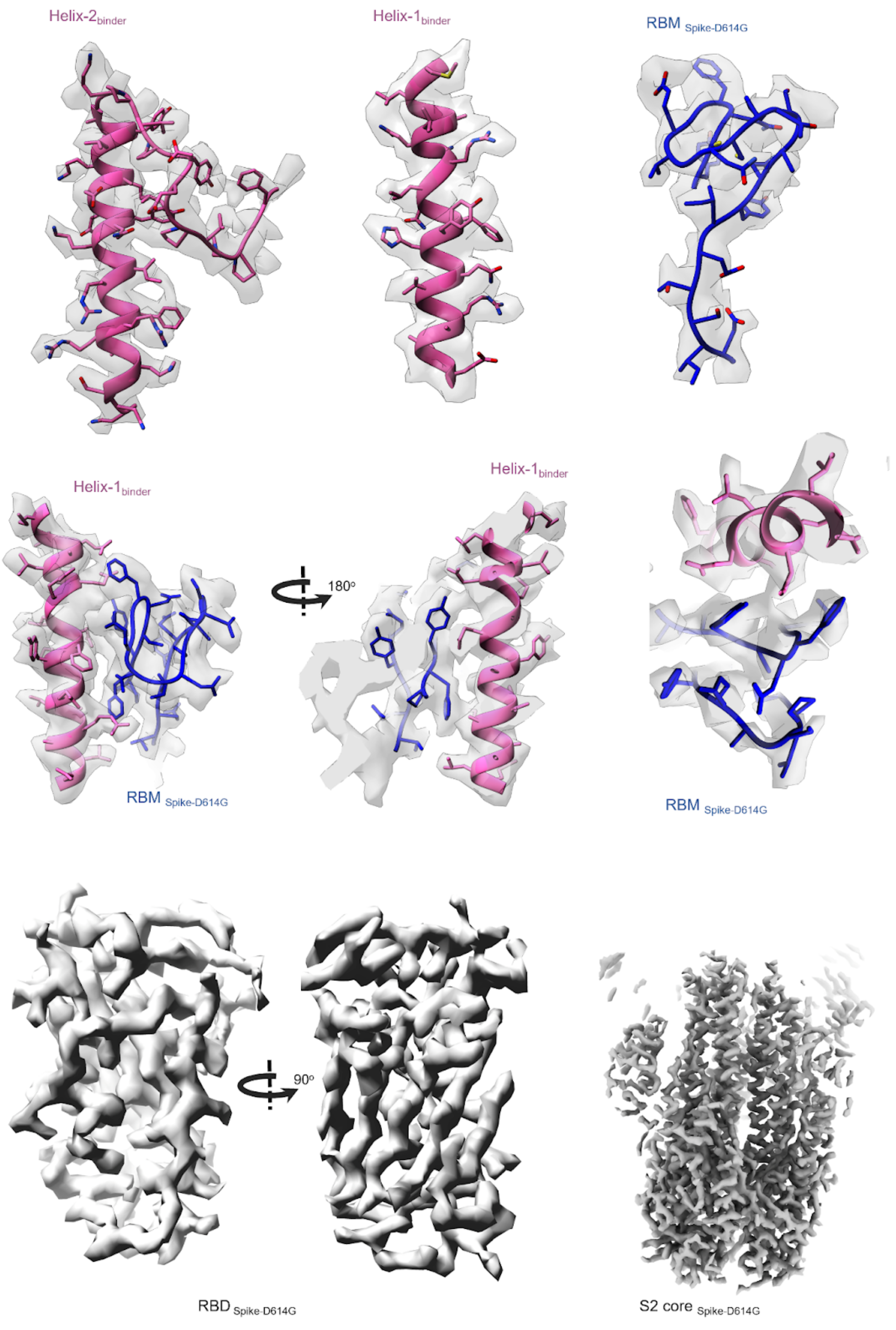

Supplementary Figure S12: Highlights of the Cryo-EM densities of DBR3_03 with D614G spike. Cryo-EM densities are shown as surfaces. RBM (receptor binding motif) in blue with DBR3_03 in pink. The atomic model is shown as stick or ribbon representation.

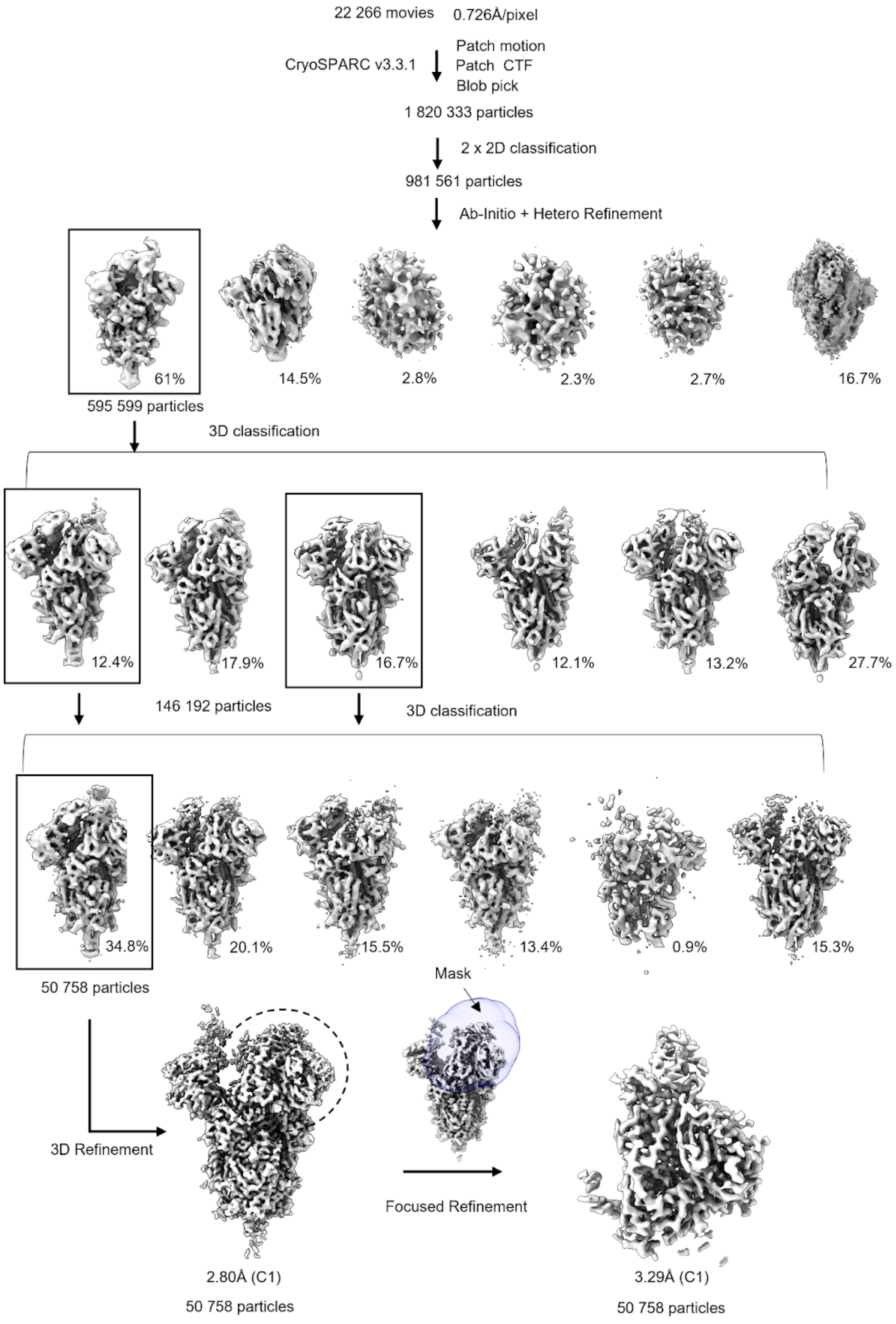

Supplementary Figure S13: Cryo-EM data processing of the Omicron Spike-DBR3_03 complex. Image processing workflows performed in CryoSPARC.

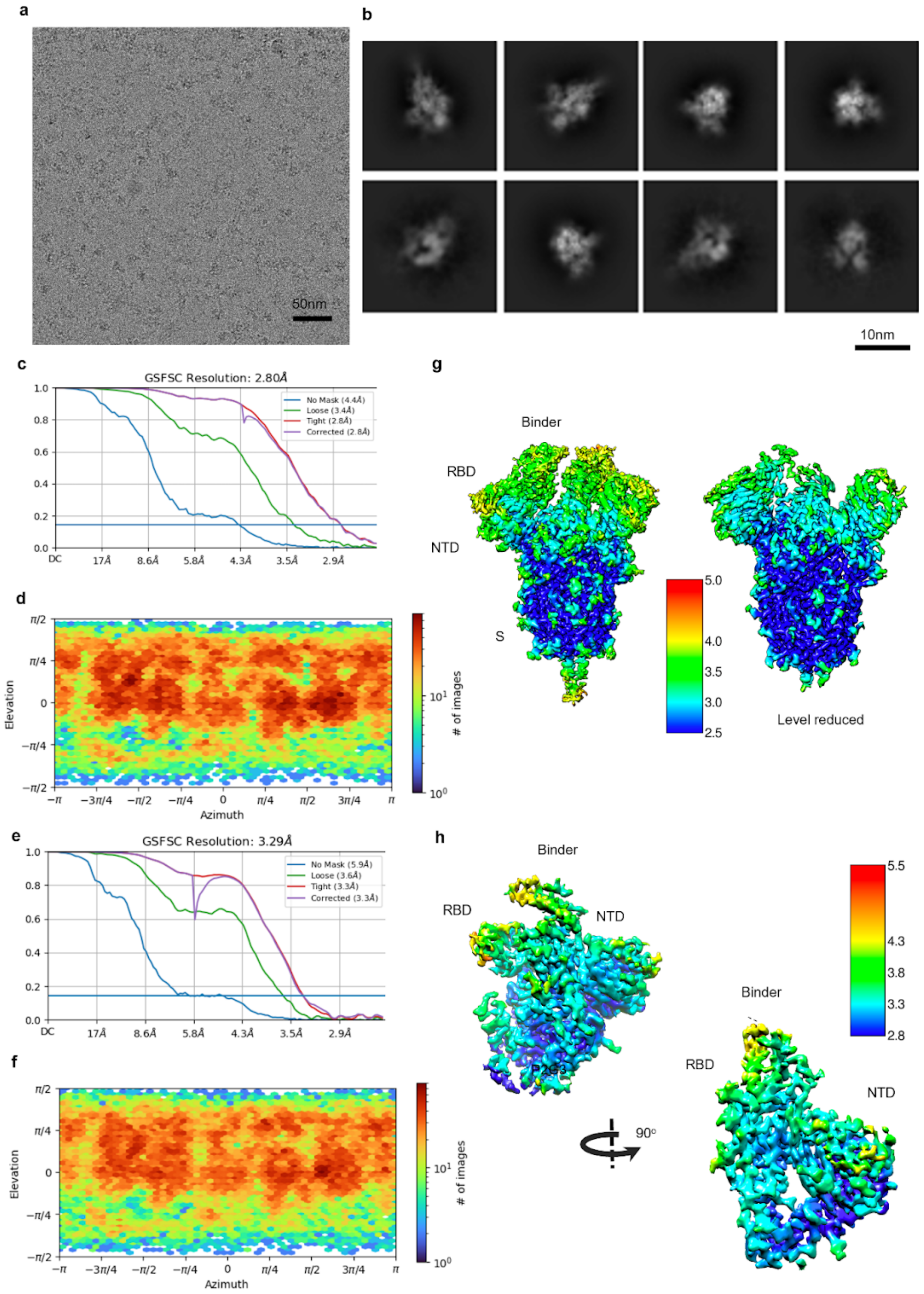

Supplementary Figure S14: Details of Cryo-EM data processing for Omicron Spike-DBR3_03 complex. a, A representative Cryo-EM micrograph for the D614G Spike-binder complex. 22,266 micrographs of such similar quality were acquired for this complex. **b,** The representative 2D classes of the omicron Spike-binder complex. **c,** Direction distribution of the particle alignment and **d,** FSC curves of the final overall map. **e,** Direction distribution and **f,** FSC curves of the locally refined map. **g,h,** Local resolution distribution of the overall and focused refined maps.

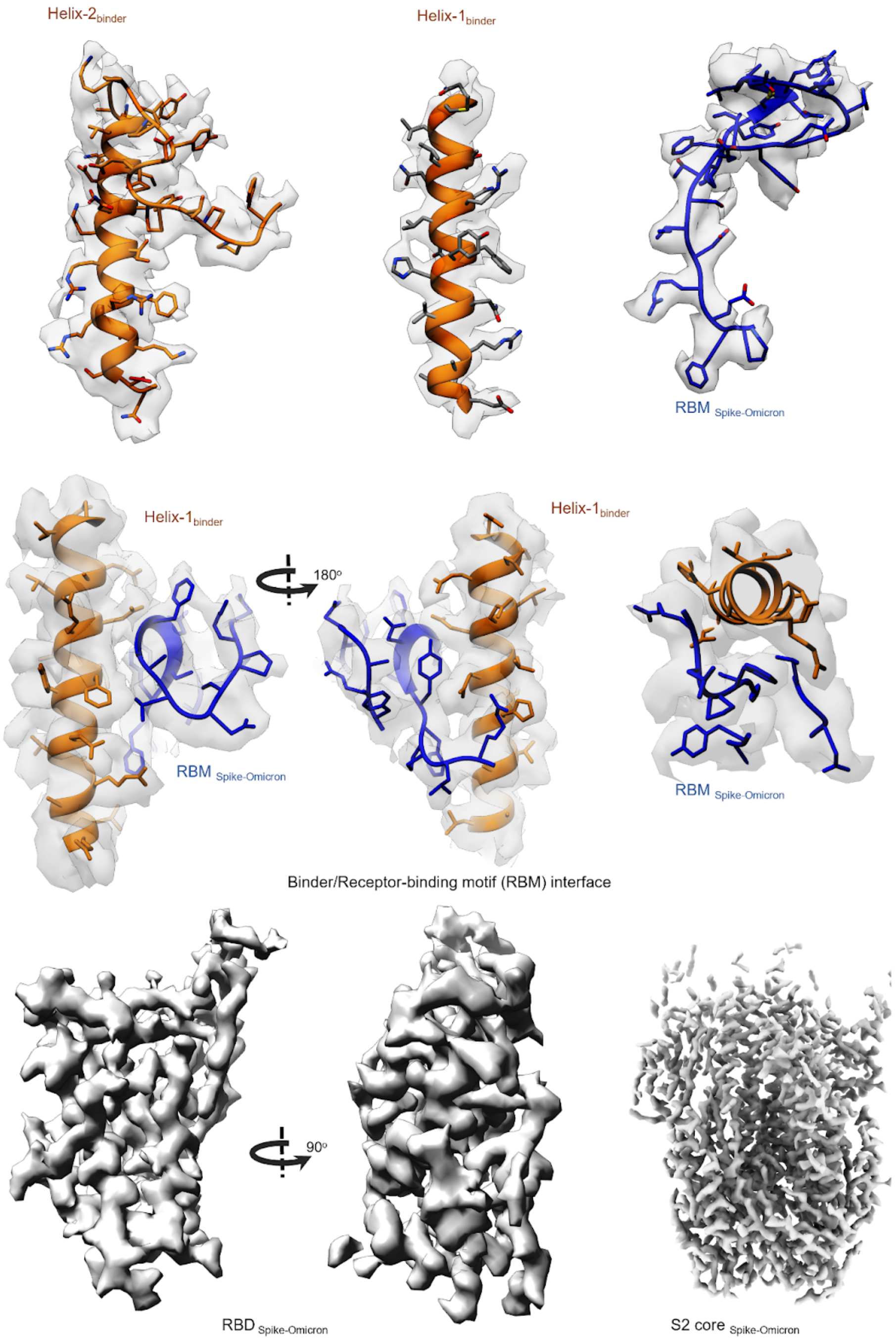

Supplementary Figure S15: Highlights of the cryo-EM densities of DBR3_03 with Omicron spike. Cryo-EM densities are shown as surfaces. RBM (receptor binding motif) in blue with DBR3_03 in orange. The atomic model is rendered as stick or ribbon representation.

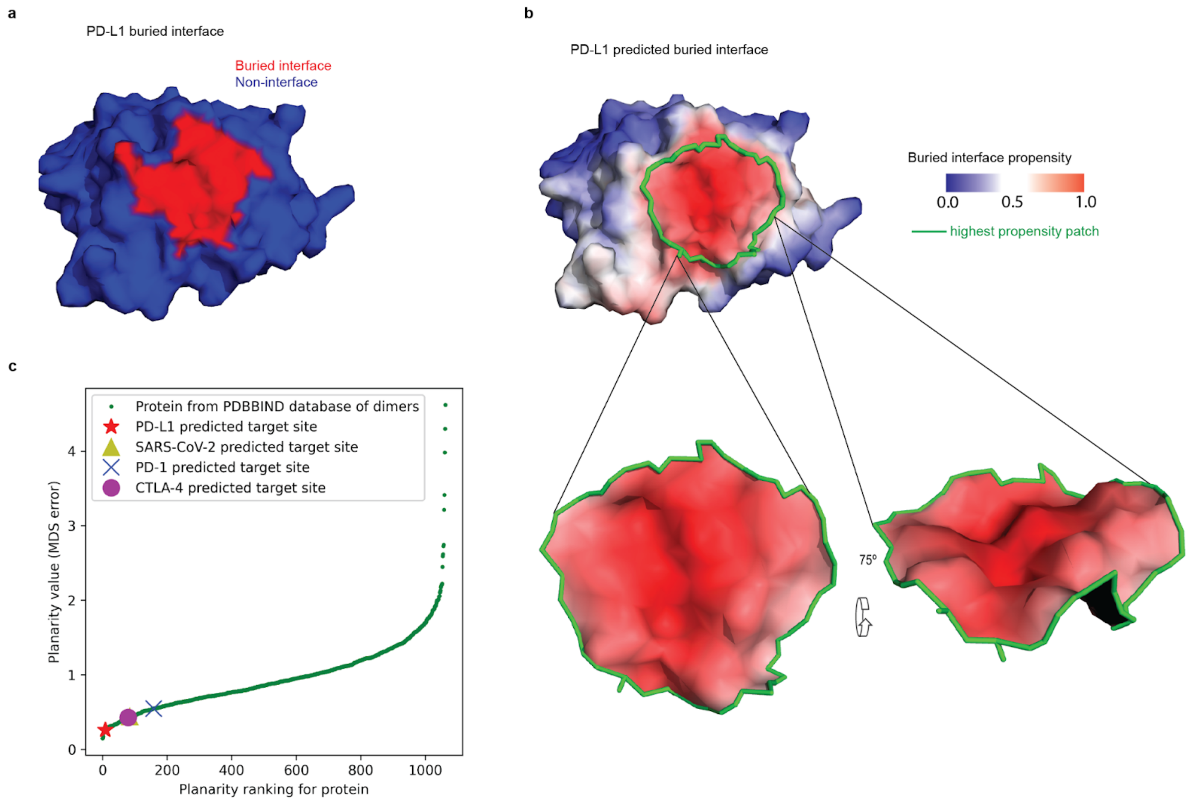

Supplementary Figure S16: Planarity of the targeted interface sites. a, Buried interface on PD-L1 upon complex formation with PD-1. **b**, (Top) PD-L1 predicted buried interface, with selected target patch marked with a green contour. (bottom) View of the selected target patch to show its planarity. **c**, Plotting of the planarity of each of 1068 dimeric protein interfaces. Y-axis: error in multidimensional scaling when flattening the patch from 3D to 2D. X-axis: ranking of each protein according to the planarity value with respect to the dataset of 1068 dimeric protein interfaces. The PD-L1 interface targeted in this work is marked with a red star, SARS-CoV-2 with a gold triangle, PD-1 with a blue X, and CTLA-4 with a magenta circle.

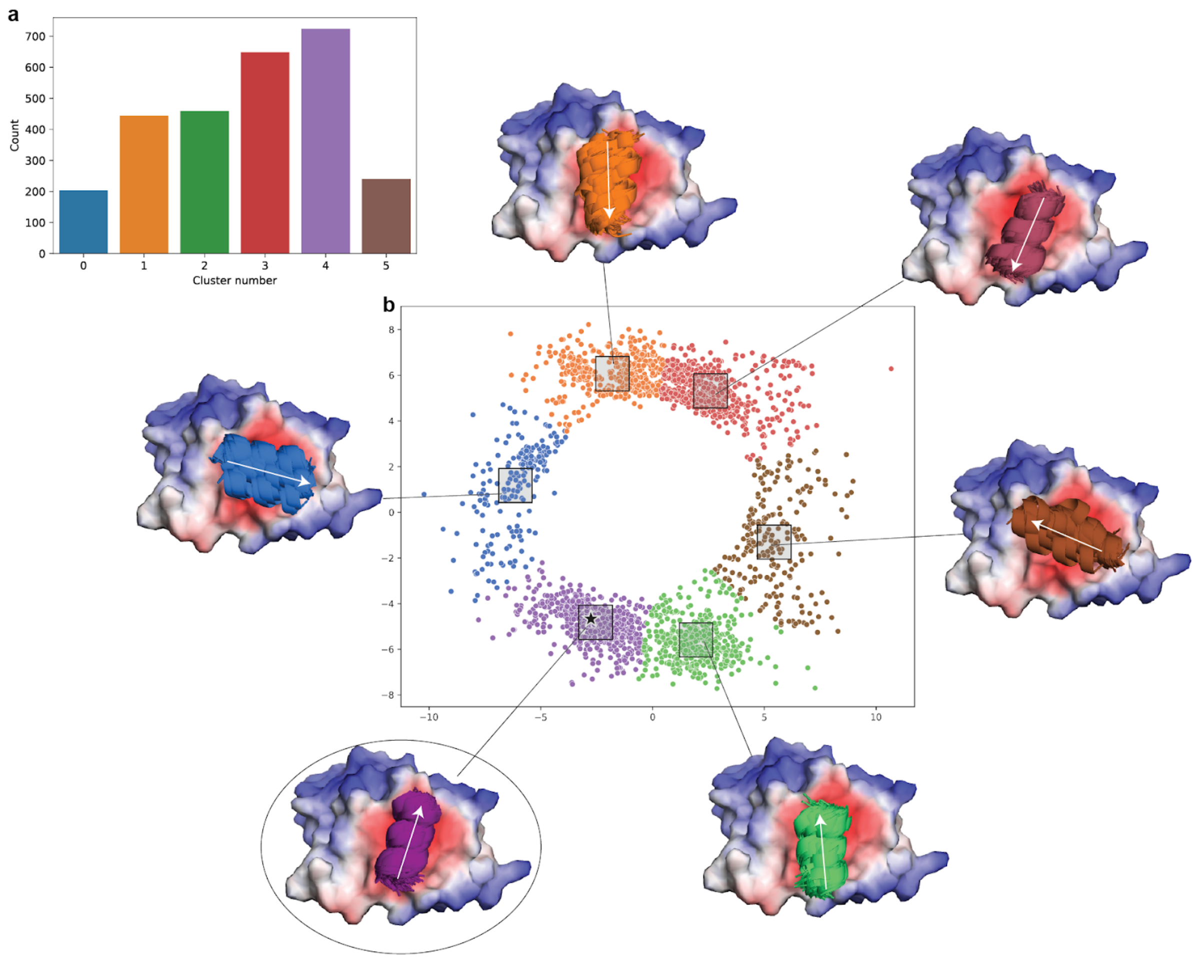

Supplementary Figure S17: Clusters of putative binding seeds identified by MaSIF-seed docked on the PD-L1 surface (PDB ID: 5JDS). 140 million patches from ~250,000 helices extracted from the PDB were compared and docked to the predicted interface in PD-L1 using MaSIF-seed. The top scoring seeds were selected for further processing. Twelve-amino acid fragments of these seeds that occupied the largest buried surface were then clustered using metric multidimensional scaling of all pairwise RMSDs between all seeds. **a,** Histogram of clusters, showing the prevalence of each orientation. **b,** Binding seed clusters in the multidimensional scaling plot. A box is drawn around the center of each cluster and the picture shows the selected helix orientation for all points inside the box. The circled binding seed cluster shows the helix orientation of the seed used for the PD-L1 designs. A star symbol shows the PD-L1 seed used for the designs.

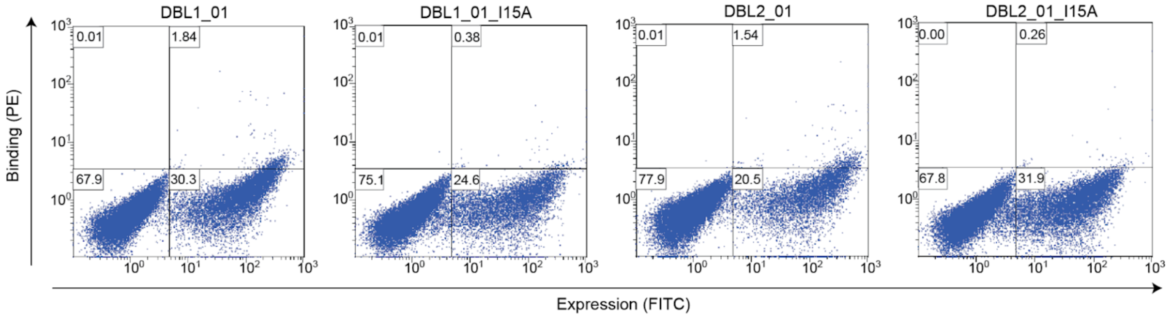

Supplementary Figure S18: Binding signals of initial PD-L1 binder designs. Binding measured on the surface of yeast with 15 μM PD-L1-Fc. Comparison of DBL1_01 and DBL2_01 with corresponding interface mutants.

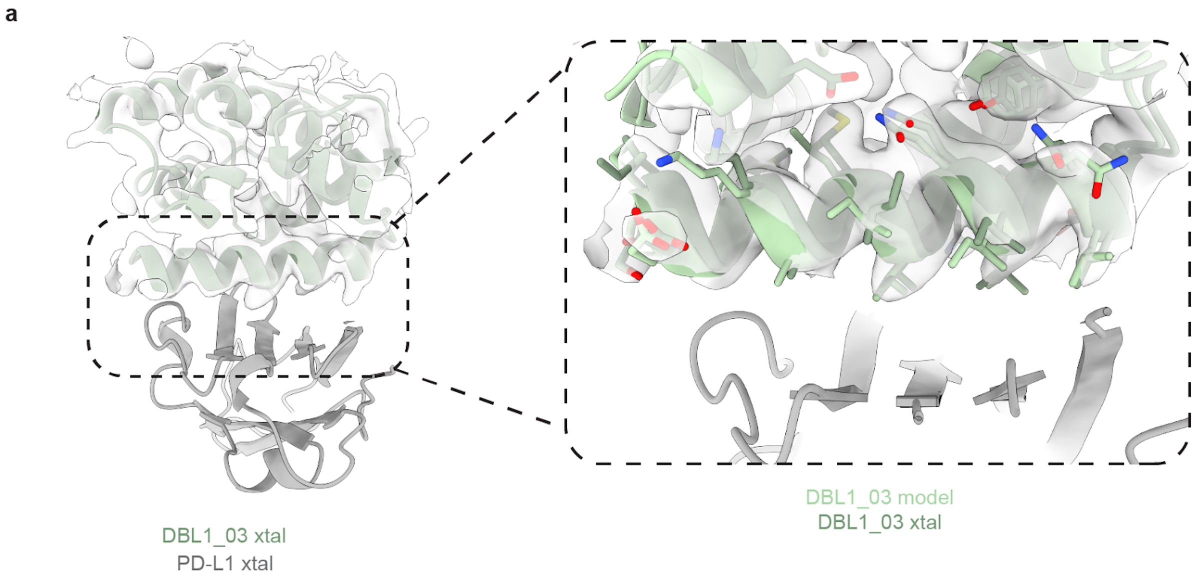

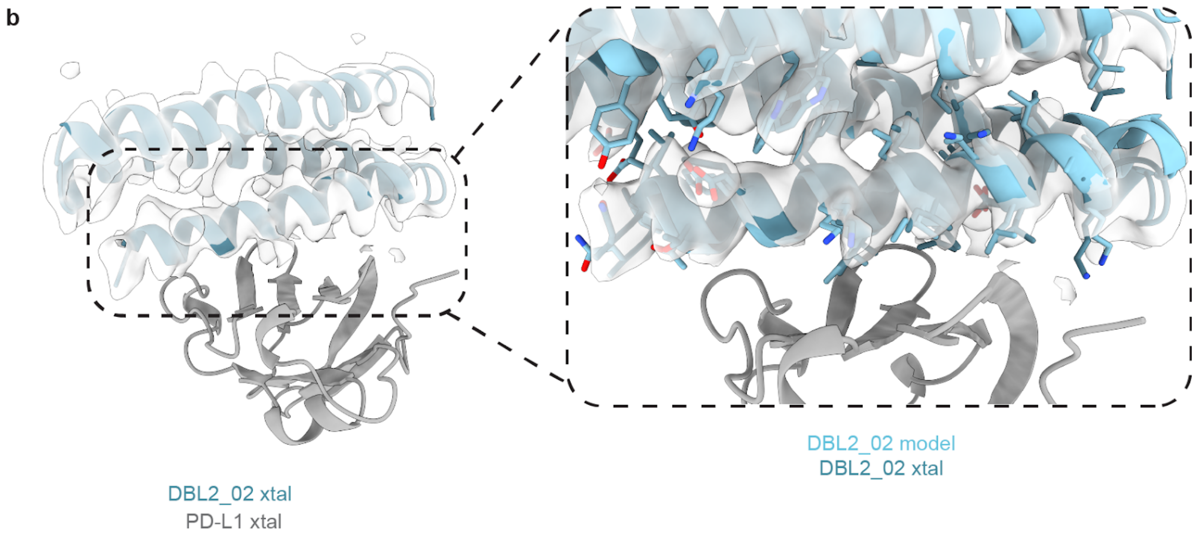

Supplementary Figure S19: Electron density map of the crystalized DBL1_03 and DBL2_02. **a**, Crystal structure of DBL1_03 (green) in complex with PD-L1 (gray). Refined 2mFo-mFc electron density map of the binder, contoured at 1.0σ, is rendered as a white surface **b**, Crystal structure of DBL2_02 (blue) in complex with PD-L1 (gray). Refined 2mFo-mFc electron density map of the binder, contoured at 1.0σ, is rendered as a white surface

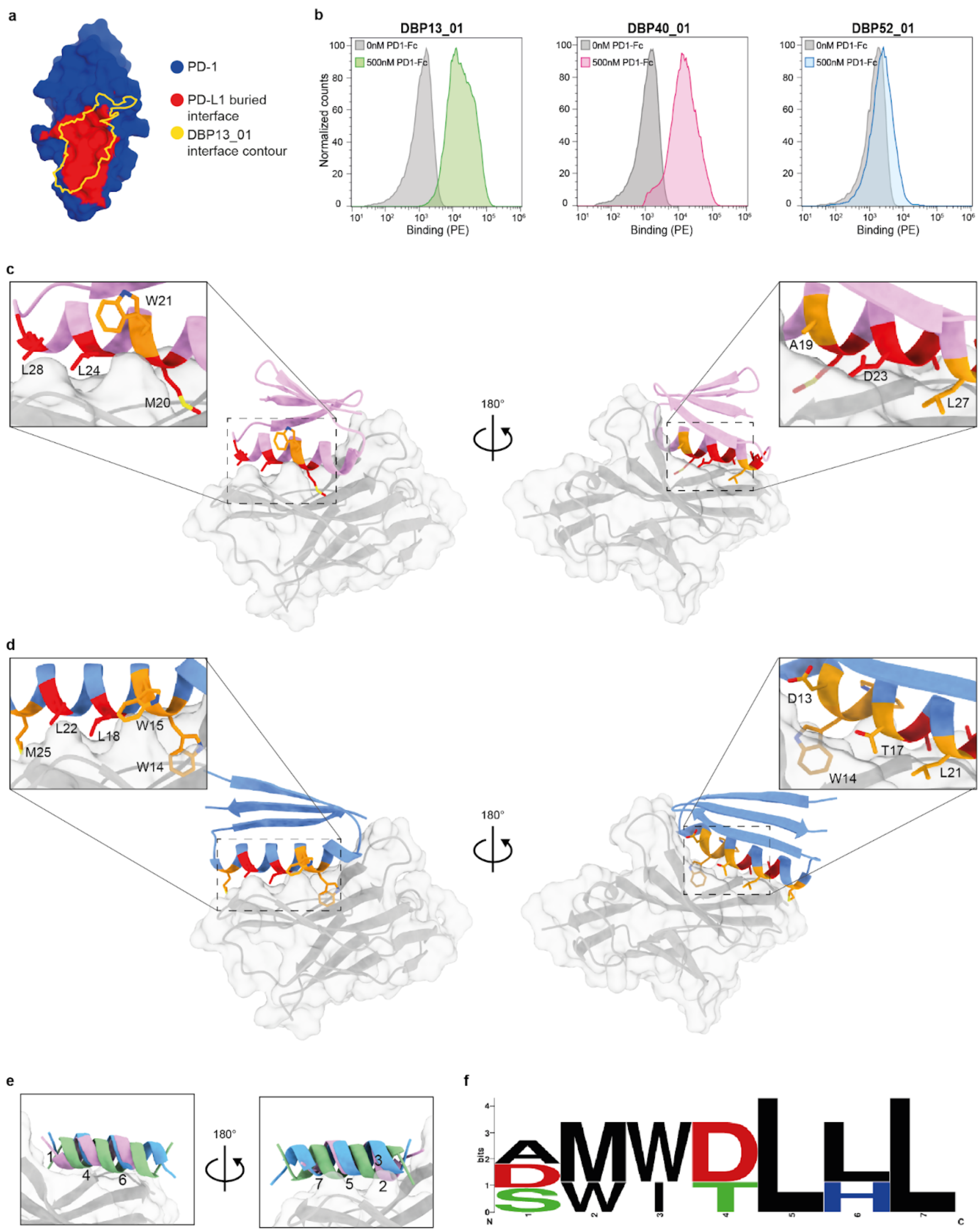

Supplementary Figure S20: Overview and comparison between PD-1 binders. a, PD-1 surface (blue) with the region targeted by PD-L1 (red) and the overlapping region targeted by DBP13_01 (yellow contour). **b**, Histograms of the binding signal (PE) measured on 3 yeast clones displaying designed binders against PD-1. Yeast cells were labeled with 500 nM PD-1-Fc (coloured) or secondary antibodies only (gray, negative control). **c-d**, Overview and close-up of DBP40_01 (a, pink) and DBP52_01 (b, blue) models in complex with PD-1 (gray). Interface seed residues similar to DBP13_01 are highlighted in red, while residues that are different are highlighted in orange. **e,** Seeds used to design DBP13_01 (green), DBP40_01 (pink) and DBP52_01 (blue) aligned with interface residues numbered. **f,** Sequence logo of the seed interface residues for the three PD-1 binders as numbered in e.

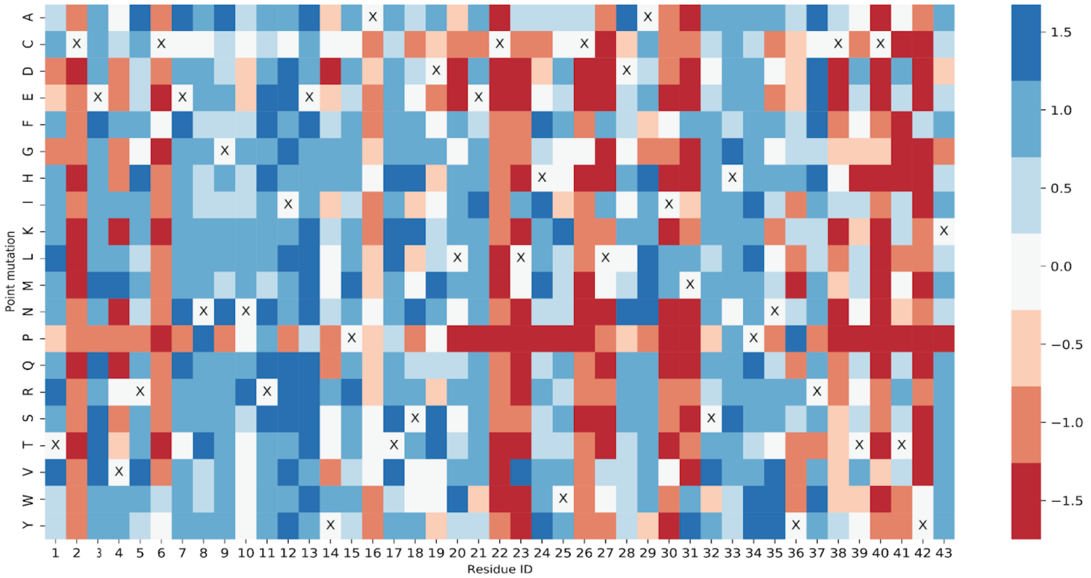

Supplementary Figure S21: SSM of DBP13_01. Heatmap covering all positions of DBP13_01. Yeast displaying point mutants were analyzed by flow cytometry and subsequently binding and non-binding populations were sorted. For each mutation the log-ratio between the enrichment in binding versus non-binding populations was computed. Mutations in red highlight a deleterious effect on binding, while mutations in blue indicate an enrichment on the binding population.

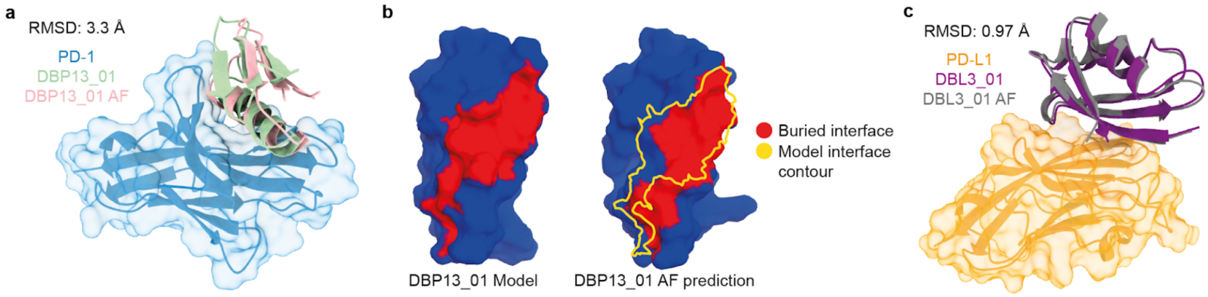

Supplementary Figure S22: AF structure prediction of DBP13_01 in complex with PD-1. a, Comparison of the DBP13_01 computational model (green) and the AlphaFold multimer (AF) prediction (red) on the surface of PD-1 (blue). **b**, Buried interfaces in both DBP13_01 model (left) and AF prediction (right) are shown in red with an overlap yellow, a yellow contour of the footprint of the original model is shown for ease of comparison. **c**, Comparison of the DBL3_01 computational model (purple) and the AlphaFold Multimer (AF) prediction (gray) on the surface of PD-L1 (orange).

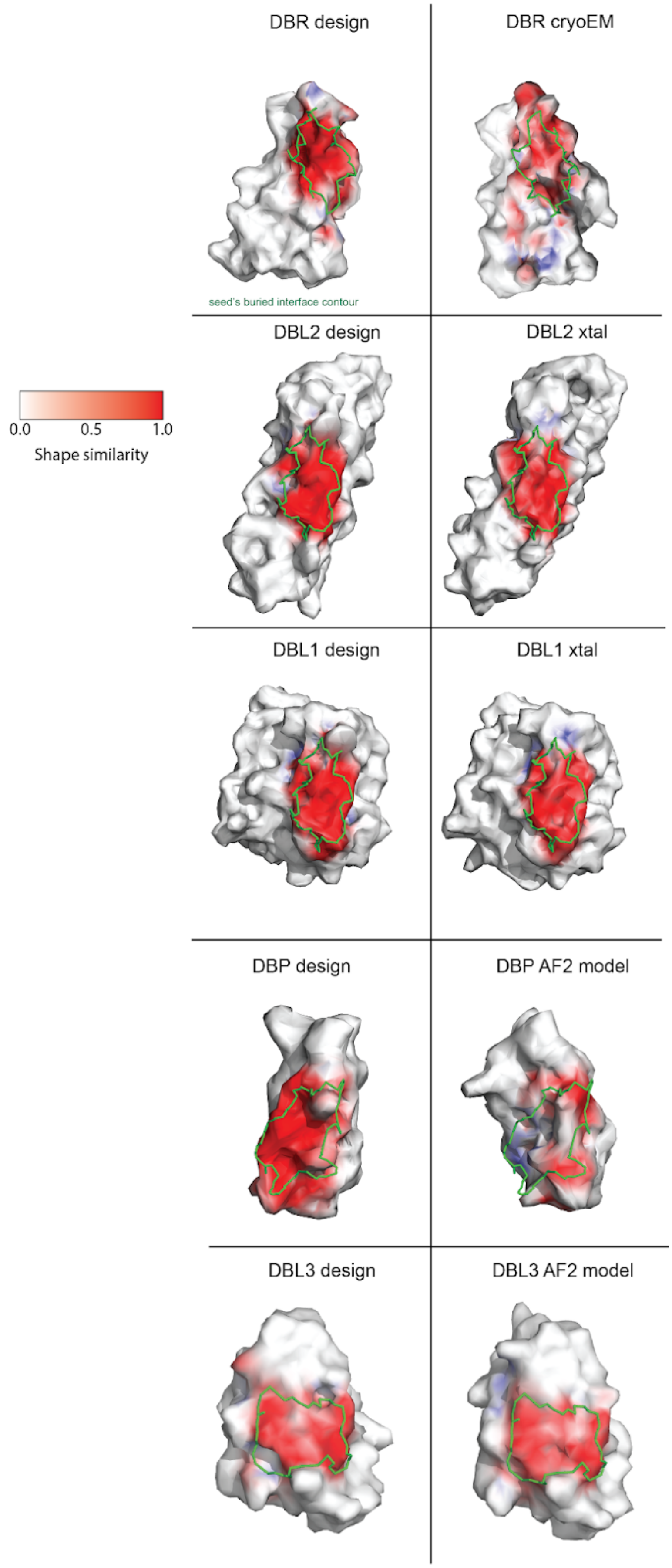
DBR design
DBR cryoEM
seed's buried interface contour
DBL2 design
DBL2 xtal
0.0
0.5
1.0
Shape similarity
DBL1 design
DBL1 xtal
DBP design
DBP AF2 model
DBL3 design
DBL3 AF2 model

Supplementary Figure S23: Surface similarity of the computational designs, experimentally solved structures or AF models relative to initial binding seeds. Each complex was aligned to the target protein (RBD, PD-L1 and PD-1), and the surface similarity of the computational design, the experimental structure or AF model to the binding seed is shown in a gradient from white to red. The buried surface area of the initial binding seed is shown by a green contour. The surface similarity was calculated in the same way as shape complementarity but normal vectors are not inverted during the process, i.e. the normal vectors for both surfaces point outwards of the molecular surface. Briefly, pairs of nearest vertices between the surface of the design or structure/model and the initial binding seed were computed based on the nearest neighbor of the aligned model. The shape similarity was evaluated by computing the dot product of the vertex pairs normal vectors yielding the enclosed angle and scaling it with the distance of the vertex pair. The resulting values are colored in a gradient from white to red and range from 0 (colored in white) indicating no similarity, to 1 (colored in red) indicating high similarity.

Supplementary Table 1: *Extended Benchmark of MaSIF-seed against other docking methods in recovering the native binder in the correct conformation from co-crystal structures for 31 helix-receptor complexes or 83 non-helix seed-receptor complexes, discriminating between 1000 decoys.* [a]*Benchmarked method.* [b-d]*Number of receptors for which the method recovered the native binding motif (<3 Å iRMSD) within the* [b]*top 1,* [c]*top 10, and* [d]*top 100 results.* [e]*Number of receptors for which the method did not recover the native binding motif in the top 100 results.* [f]*Average running time in minutes, excluding pre-computation time.*

	**Method**[a]	**# in top 1**[b]	**# in top 10**[c]	**# in top 100**[d]	**>100**[e]	**Avg time (m)**[f]
**Helical seeds**	MaSIF-seed	18	18	20	11	15
	PatchDock+MaSIF-site	3	5	11	20	86
	ZDOCK	3	4	8	23	2715
	ZDOCK+ MaSIF-site	1	6	10	21	2485
	ZDOCK+ ZRank2	6	12	21	10	2946
	ZDOCK+ ZRank2+ MaSIF-site	5	11	19	12	2710
**Non-helical seeds**	MaSIF-seed	41	47	49	34	118
	ZDock	7	9	22	61	2206
	ZDock+ ZRank2	21	33	45	38	2400

Supplementary Table 2: Sequences of the designed proteins.

Design	Sequence	# of mutations from WT	# of mutations from design_01	Mutations
DBL1 native scaffold (PDB ID: 3S0D)	MTIEELKTRLHTEQSVCKTETGIDQQKANDVIEGNIDVEDKKVQLYCECILKNFNILDKNNVFKPQGIKAVMELLIDENSVKQLVSDCSTISEENPHLKASKLVQCVSKYKTMKSVDFL			
DBL1_01	TSIESLKWTLIVEQILCQLDTGIDQQKANDVIEGNIDVEDKKVQLYCECILKAFHILDKNNVFKPQGIKAVMELLIDENSVKQLVSDCSTISEENPHLKASKLVQCVSKYKTMKSVDFL	14		M1T, T2S, E5S, T8W, R9T, H11I, T12V, S15I, V16L, K18Q, T19L, E20D, N53A, N55H
DBL1_02	SSIESLKWSLIVQQILCQLETGIDQQKANDVIEGNIDVEDKKVQLYCECILKQFHILDKNNVFKPQGIKAVMELLIDENSVKQLVSDCSTISEENPHLKASKLVQCVSKYKTMKSVDFL	14	5	T1S, T9S, E13Q, D20E, A53Q
DBL1_03	SSIESMKWSMIVQQILCQLETGIDQQKANDVIEGNIDVEDKKVQLYCECILKQFHILDKNNVFKPQGIKAVMELLIDENSVKQLVSDCSTISEENPHLKASKLMQCISKYKTWKSFDFL	20	11	L6M, L10M, V104M, V107I, M113W, V116F
DBL1_04	SSIESMKWSMIRQQILCQLETGIDQQKANDVIEGNIDVEDKKVQLYCECILKQFHILDKNNVFKPQGIKAVMELLIDENSVKQLVSDCSTISEENPHLKASKLMQCISKYKTWKSFDFL	21	14	E4T, W8N, Q18R
DBL2 native scaffold (PDB ID: 3ONJ)	SLLISYESDFKTTLEQAKASLAEAPSQPLSQRNTTLKHVEQQQDELFDLLDQMDVEVNNSIGDASERATYKAKLREWKKTIQSDIKRPLQSLVDSGD			
DBL2_01	SLLESYEWSFIVQLILAKLELAYAPSQPLSQRNEQLKRVEQQQDQLFDLLDQMDVEVNNSIGDASERATYKAKLREWKKTIQSDIKR	15		I4E, S8W, D9S, K11I, T12V, T13Q, E15I, Q16L, A19L, S20E, E23Y, T34E, T35Q,

	PLQSLVDSGD			H38R, E45Q
DBL2_02	NLLESYEWSFKVQLILAKLELAKAPSQPLSQRNEELKRVEQRQDRLFDLLDQMDVEVNNSIGDASERATYKAKLREWKKTIQSDIKRPLQSLVDSGD	16	6	S1N, I11K, Y23K, Q35E, Q42R, Q45R
DBL2_03	NLLTSYEGSFKIQLILAKLELAKAPSQPLSQRNEELKRVEQRQDRLFDLLDQMDVEVNNSIGDASERATYKAKLREWKKTIQSDIKRPLQSLVDSGD	16	9	E4T, W8G, V12I
DBL2_04	NLLRSYENSFKIQLILAKLELAHAPSQPLSQRNEELKRVEQRQDRLFDLLDQMDVEVNNSIGDASERATYKAKLREWKKTIQSDIKRPLQSLVDSGD	16	9	T4R, G8N, K23H
DBR_01	STNMLEALQQRLHKYAAVVSRAALENNSGKARRFGRIVKQYEDAIKLYKAGKPVPYDELPVPPGFG	8		E13H, Q16A, S17A, E19V, A20S, A21R, K23A, A24L
DBR_02	STNMLEALQQRLQFYFGVVSRAALENNSGKARRFGRIVKQYEDAIKLYKAGKPVPYDELPVPPGFG	9	4	H13Q, K14F, A16F, A17G
DBR_03	STNMLEALQQRLQFYHGQVARAALENNSGKARRFGRIVKQYEDAIKLYKAGKPVPYDELPVPPGFG	9	6	F16H, V18Q, S20A
DBR_03_KO	STNMLEALQQRLQFYHRQVRRAALENNSGKARRFGRIVKQYEDAIKLYKAGKPVPYDELPVPPGFG	9		G17R, A20R
DBP13_01	TCEVRCENGNRIEYPATSDLECLHWCLDAIMSHPNYRCTCTHK	10		Q10N, E20L, E23L, R24H, R27L, K28D, K30I, K31M, E32S, F33H
DBP13_01 (native scaffold)	TCEVRCENGQRIEYPATSDEECERWCRKAKKEFPNYRCTCTHK			
DBP40_01	SQVTWNGVTVTNDNPSQSAMWADLIALLYQGEVRVKDGRWEIH	12		I1S, F12N, E16S, E17Q, A18S, E19A, K20M, Y21W, K23D, K24L, K27L, E28L
DBP40_01 (native scaffold)	IQVTWNGVTVTFDNPEEAEKYAKKIAKEYQGEVRVKDGRWEIH			

DBP52_01	QKETRHCSGRSCDWWATLWCLLCAMKGKRVRCRQHGQQVEVQCDK	13		Q10R, R11S, E13D, Q14W, E15W, R17T, R18L, E21L, E22L, K24A, K25M, K34Q, N37Q
DBP52_01 (native scaffold)	QKETRHCSGQRCEQEARRWCEECKKKGKRVRCRKHGNQVEVQCDK			
DBL3_01	AQTILLRLEGMDCTSCASSIERAIAKVPGVQQCIVFFEMNLAIVTYHGETTPQILTDAVERAGYHARVS	11		N5L, Q7R, S32Q, Q34I, N36F, A38E, L39M, E40N, Q41L, V43I, S45T
DBL3_02	AQTILLRLEGMDSTSSASSIERAIAKVPGVQQCIVFFEMNLAIVTYHGETTPQILTDAVERAGYHARVS	13	2	N5L, Q7R, C13S, C16S, S32Q, Q34I, N36F, A38E, L39M, E40N, Q41L, V43I, S45T
DBL3_01 (Native scaffold)	AQTINLQLEGMDCTSCASSIERAIAKVPGVQSCQVNFALEQAVVSYHGETTPQILTDAVERAGYHARVL			
DBL4_01	PDRWLLRIEIPADIAANEALKVRLLETEGVKIVFINESSHAALVIIDSKVTNRFEVEQAIRQ	12		Y2D, V3R, S4W, S5L, E32I, L34F, A36N, E38S, E39S, S41A, Y43L, K45I
DBL4_01 (Native scaffold)	PYVSSLRIEIPADIAANEALKVRLLETEGVKEVLIAEEEHSAYVKIDSKVTNRFEVEQAIRQA			
DBC2_01	AFITIMDGEEKARKYAKMLKKQNLKVIVLMANGKWIIYAK	11		K1A, T3I, T5I, E25K, H27I, R29L, V30M, E31A, V36I, T38Y, E40K
DBC2_01 (Native scaffold)	KFTTTMDGEEKARKYAKMLKKQNLEVHVRVENGKWVITAE			

Supplementary Table 3: SARS-CoV-2 variant mutations.

Variant (graph label in bold)	Mutations	EC_{50} of DBR3_03 with variant:
D614G / **WT**	D614G	6.61e-8 g/mL
B.1.1.7 / **Alpha**	Δ69-70, Δ144, N501Y, A570D, D614G, P681H, T716I, S982A, D1118H	6.56e-8 g/mL
B.1.351 / **Beta**	L18F, D80A, D215G, Δ242-244, R246I, K417N, E484K, N501Y, D614G, A701V	6.11e-7 g/mL
B.11.28.1 / **Gamma**	L18F, T20N, P26S, D138Y, R190S, K417T, E484K, N501Y, D614G, H655Y, T1027I, V1176F	6.76e-7 g/mL
B.1.526 / **Iota**	L5F, T95I, D253G, E484K, D614G, A701V	4.13e-7 g/mL
B.1.617.1 / **Kappa**	E154K, L452R, E484Q, D614G, P681R, Q1071H	NA
B.1.617.2 / **Delta**	T19R, Δ156-157, R158G, L452R, T478K, D614G, P681R, D950N	NA
**Lambda**	G75V, T76I, R246N, Δ247-253, L452Q, F490S, D614G, T859N	NA
**Omicron BA.1**	A67V, Δ69-70, T95I, G142D, Δ143-145, Δ211, L212I, ins214EPE, G339D, S371L, S373P, S375F, K417N, N440K, G446S, S477N, T478K, E484A, Q493K, G496S, Q498R, N501Y, Y505H, T547K,D614G, H655Y, N679K, P681H, N764K, D796Y, N856K, Q954H, N969K, L981F	5.68e-8 g/mL
**Omicron BA.2**	T19I, Δ24-26, A27S, G142D, V213G G339D, S371F, S373P, S375F, T376A, D405N, R408S, K417N, N440K, S477N, T478K, E484A, Q493R, Q498R, N501Y, Y505H, D614G, H655Y, N679K, P681H, N764K, D796Y, Q954H, N969K	4.35e-8 g/mL

Supplementary Table 4: Summary of binding candidates obtained after deep sequencing with the optimized design pipeline. Binding seeds were helical (H) or strand (E). Deep sequencing data comprises reads from the non-binding (Neg reads) and binding population (Pos reads). The enrichment score is calculated based on the logarithm of the ratio between positive and negative reads. Computational models of the complexes were predicted by AlphaFold Multimer (AF) and aligned with respect to the target. Binding signals detected on the surface of yeast (at 500 nM ligand) were categorized as negative(-), marginal (-/+), weak (+), moderate (++) or high (+++). Marginal and weak binding signals were not further characterized (Competition assay, knock-out mutants and negative controls).

**Design**	**Target**	**Scaffold origin**	**Scaffold name**	**Motif**	**Neg reads**	**Pos reads**	**Enrichment**	**AF RMSD [Å]**	**Binding**	**Competition**	**Knock-out**	**Neg Ctrl**
DBP13_01	PD-1	Miniprotein	EEHE_2.1_02	H	776	277011	2,5526	3,3	+++	OK	OK	OK
DBP40_01	PD-1	Miniprotein	EEHEE_rd4_0499	H	15	18596	3,0933	7,1	+++	OK	Not tested	OK
DBP48_01	PD-1	Miniprotein	EHEE_rd4_0510	H	225	2354	1,0196	16,9	-	N/A	N/A	N/A
DBP52_01	PD-1	Miniprotein	EHEE_1.7_09	H	12	934	1,8912	15,5	+	Not tested	Not tested	Not tested
DBC1_01	CTLA-4	Miniprotein	EEHE_2.1_06	E	3	1107	2,567	6,2	-	N/A	N/A	N/A
DBC2_01	CTLA-4	Miniprotein	EHEE_rd4_0923	E	172	39697	2,3632	34,6	++	OK	OK	OK
DBC3_01	CTLA-4	Miniprotein	EHEE_rd4_0042	E	31	3797	2,0881	30,4	-/+	N/A	N/A	N/A
DBC4_01	CTLA-4	Miniprotein	EHEE_rd4_0924	E	55	4018	1,8636	34,9	-/+	N/A	N/A	N/A
DBC5_01	CTLA-4	Miniprotein	EHEE_rd4_0448	E	35	1203	1,5362	24,3	-	N/A	N/A	N/A
DBC6_01	CTLA-4	Miniprotein	EHEE_rd4_0357	E	109	1281	1,0701	22,4	-	N/A	N/A	N/A
DBC7_01	CTLA-4	Miniprotein	EHEE_rd4_0924	E	114	1333	1,0679	38,4	-	N/A	N/A	N/A
DBC8_01	CTLA-4	Miniprotein	EHEE_rd4_0636	E	3	2162	2,8577	33,6	-	N/A	N/A	N/A
DBC9_01	CTLA-4	Miniprotein	EHEE_rd4_0811	E	44	1166	1,4232	32,2	-	N/A	N/A	N/A
DBL3_01	PD-L1	PDB	4A48 (B)	E	654	44391	1,8317	1,2	++	OK	OK	OK
DBL4_01	PD-L1	PDB	4Q2M (A)	E	306	10238	1,5245	9,1	++	OK	OK	OK
DBL5_01	PD-L1	Miniprotein	EHEE_rd4_0017	E	340	5443	1,2044	15,6	-	N/A	N/A	N/A

Supplementary Table 5: Antibodies used in flow cytometry experiments.

Antibody	Catalog number	Supplier	Dilution
Anti-HA, FITC	A190-138F	Bethyl	1:100
Anti-V5 mouse	MA5-15253	Invitrogen	1:333
Anti-mouse, FITC	F0257	Sigma	1:100
Anti-His, PE	130-120-787	Miltenyi Biotec	1:50
Anti-Myc, FITC	SAB4700448	Sigma	1:100
Anti-human IgG, PE	12-4998-82	Invitrogen	1:100
Anti-Human IgG, R-PE	109-117-008	Jackson ImmunoResearch	1:100

Supplementary Table 6: Primer sequences.

Library:	Primer name:	Primer sequence:
DBR_01	5vny_rev_1	CAGACGTTGCTGTAGGGCCTCAAGCATGTTCGTGCTGCTAGCAGCGTAGTCTGGAACG
DBR_01	5vny_fwd_2a	GAGGCCCTACAGCAACGTCTGCWMARATACKYCRBRGTABNARSCNNSGCGGSACTTGAGAATAATAGTGGAAAAGCAAGAAGATTTGGCAGGATC
DBR_01	5vny_fwd_2b	GAGGCCCTACAGCAACGTCTGCWMYWCTACKYCRBRGTABNARSCNNSGCGGSACTTGAGAATAATAGTGGAAAAGCAAGAAGATTTGGCAGGATC
DBR_01	5vny_rev_3	ACAGGTTTTCCAGCTTTATACAACTTAATTGCGTCCTCGTATTGTTTAACGATCCTGCCAAATCTTCTTGCTTT
DBR_01	5vny_fwd_4	ATTAAGTTGTATAAAGCTGGAAAACCTGTACCATACGACGAACTACCTGTCCCGCCAGGATTCGGCGGATCCCAGGAACTGACAACTATATG
DBL1_L1	3S0D_fw1	GCCTTAGCTCAACCGGTTATTTCTACTACCGTCGGTTCCGCTGCAGAAGGCTCTTTGGACAAGAG
DBL1_L1	3S0D_rev1	GCTAGCAGCGTAGTCTGGAACGTCGTATGGGTAAGCTTCTCTCTTGTCCAAAGAGCCTTCT
DBL1_L1	3S0D_fw3a	CCAGACTACGCTGCTAGCHHCTMCATTSWAAGTTTGAAGTGGAVCTTAWTCRTASAACAAATTVTATGTCAACTTBWCACGGGGATTGACCAGCA
DBL1_L1	3S0D_ fw3b	CCAGACTACGCTGCTAGCHHCTMCATTSWAAGTTTGAAGTGGAVCTTAWTCRTASAACAAATTVTATGTCAACTTGAAACGGGGATTGACCAGCA
DBL1_L1	3S0D_ fw3c	CCAGACTACGCTGCTAGCHHCTMCATTSWAAGTTTGAAGTGGAVCTTAWTCRTATGGCAAATTVTATGTCAACTTBWCACGGGGATTGACCAGCA
DBL1_L1	3S0D_ fw3d	CCAGACTACGCTGCTAGCHHCTMCATTSWAAGTTTGAAGTGGAVCTTAWTCRTATGGCAAATTVTATGTCAACTTGAAACGGGGATTGACCAGCA
DBL1_L1	3S0D_ fw3e	CAGACTACGCTGCTAGCHHCTMCATTSWAAGTTTGAAGTGGAVCTTAWTCRTAWWCCAAATTVTATGTCAACTTBWCACGGGGATTGACCAGCA
DBL1_L1	3S0D_ fw3f	CCAGACTACGCTGCTAGCHHCTMCATTSWAAGTTTGAAGTGGAVCTTAWTCRTAWWCCAAATTVTATGTCAACTTGAAACGGGGATTGACCAGCA
DBL1_L1	3S0D_rev4	CATTCGCAATATAGTTGGACTTTTTTGTCCTCCACGTCAATGTTCCCCTCAATCACGTCATTCGCCTTCTGCTGGTCAATCCCCGT
DBL1_L1	3S0D_fw5a	GTCCAACTATATTGCGAATGTATACTAAAASMATTCTGGATACTTGATARAAATAATGTTTTTAAGCCCCAGGGAATTAAAGC
DBL1_L1	3S0D_fw5b	GTCCAACTATATTGCGAATGTATACTAAAASMATTCYWCATACTTGATARAAATAATGTTTTTAAGCCCCAGGGAATTAAAGC

DBL1_L1	3S0D_rev6	GATATAGTGCTACAGTCGGAGACAAGCTGTTTAACGCTATTTTCATCTATTAACAGTTCCATCACAGCTTTAATTCCCTGGGGCTT
DBL1_L1	3S0D_fw7	CTCCGACTGTAGCACTATATCAGAAGAGAACCCACATCTTAAGGCCAGTAAACTGGTTCAGTGCGTGAGTAAATACAAAACCATGAAAAGCGTGG
DBL1_L1	3S0D_rev8	GAGTACGGCGTCGATTCTAAAGTTGGTGAGGGGATTTGCTCGCATATAGTTGTCAGTTCCTGGGATCCCAAGAAGTCCACGCTTTTCATGGTTTTG
DBL1_L2	3S0D_core_fw3a	CCAGACTACGCTGCTAGCTCCTCCATTGAAAGTVTGAAGTGGAGCMTGATCGTACAACAAATTCTATGTCAACT
DBL1_L2	3S0D_core_fw3b	CCAGACTACGCTGCTAGCTCCTCCATTGAAAGTVTGAAGTGGAGCTWCATCGTACAACAAATTCTATGTCAACT
DBL1_L2	3S0D_core_rev4	CGTCAATGTTCCCCTCAATCACGTCATTCGCCTTCTGCTGGTCAATCCCCGTTTCAAGTTGACATAGAATTTGTTGTACGAT
DBL1_L2	3S0D_core_fw5	TGATTGAGGGGAACATTGACGTGGAGGACAAAAAAGTCCAAC
DBL1_L2	3S0D_core_rev6a	GGCTTAAAAACATTATTTTTATCAAGTATGTGCCATTGTTTGWRCCAACATTCGCAATATAGTTGGACTT
DBL1_L2	3S0D_core_rev6b	GGCTTAAAAACATTATTTTTATCAAGTATGTGGWRTTGTTTGWRCCAACATTCGCAATATAGTTGGACTT
DBL1_L2	3S0D_core_rev6c	GGCTTAAAAACATTATTTTTATCAAGTATGTGCCATTGTTTGWRGWWACATTCGCAATATAGTTGGACTT
DBL1_L2	3S0D_core_rev6d	GGCTTAAAAACATTATTTTTATCAAGTATGTGGWRTTGTTTGWRGWWACATTCGCAATATAGTTGGACTT
DBL1_L2	3S0D_core_fw7a	CACATACTTGATAAAAATAATGTTTTTAAGCCCCAGGGAATTAAAGCTRTGATGGAACTGACTATAGATGAAAATAGCGTTAAACAGCTT
DBL1_L2	3S0D_core_fw7b	CACATACTTGATAAAAATAATGTTTTTAAGCCCCAGGGAATTAAAGCTRTGATGGAACTGMTAATAGATGAAAATAGCGTTAAACAGCTT
DBL1_L2	3S0D_core_rev8	CAGTTTACTGGCCTTAAGATGTGGGTTCTCTTCTGATATAGTGCTACAGTCGGAGACAAGCTGTTTAACGCTATTTTCATC
DBL1_L2	3S0D_core_fw9a	CACATCTTAAGGCCAGTAAACTGRYGCAGTGCVTRTMCAAGTACAAGACCTWCAAAAGCKKGGATTTCCTTGGATCCCAGGA
DBL1_L2	3S0D_core_fw9b	CACATCTTAAGGCCAGTAAACTGRYGCAGTGCVTRTMCAAGTACAAGACCTWCAAAAGCTWCGATTTCCTTGGATCCCAGGA
DBL1_L2	3S0D_core_fw9c	CACATCTTAAGGCCAGTAAACTGRYGCAGTGCVTRTMCAAGTACAAGACCWKGAAAAGCTWCGATTTCCTTGGATCCCAGGA

DBL1_L2	3S0D_core_rev10	GAGTACGGCGTCGATTCTAAAGTTGGTGAGGGGATTTGCTCGCATATAGTTGTCAGTTCCTGGGATCCAAGGAAATC
DBL2_L1	3ONJ_fw1	GCCTTAGCTCAACCGGTTATTTCTACTACCGTCGGTTCCGCTGCAGAAGGCTCTTTGGACAAGAG
DBL2_L1	3ONJ_rev2	GCTAGCAGCGTAGTCTGGAACGTCGTATGGGTAAGCTTCTCTCTTGTCCAAAGAGCCTTCTG
DBL2_L1	3ONJ_fw3a	CAGACTACGCTGCTAGCWMTCTTSDAGAGAGTTATGAATGGASCTTTRWAGTCCRATTGAWATTGGCTAAGTTGGAMCTGGCCMRGGCGCCATCACAGCC
DBL2_L1	3ONJ_fw3b	CAGACTACGCTGCTAGCWMTCTTSDAGAGAGTTATGAATGGASCTTTRWAGTCCRATTGAWATTGGCTAAGTTGGAMCTGGCCTATGCGCCATCACAGCC
DBL2_L1	3ONJ_fw3c	CAGACTACGCTGCTAGCWMTCTTSDAGAGAGTTATGAATWTASCTTTRWAGTCCRATTGAWATTGGCTAAGTTGGAMCTGGCCMRGGCGCCATCACAGCC
DBL2_L1	3ONJ_fw3d	CAGACTACGCTGCTAGCWMTCTTSDAGAGAGTTATGAATWTASCTTTRWAGTCCRATTGAWATTGGCTAAGTTGGAMCTGGCCTATGCGCCATCACAGCC
DBL2_L1	3ONJ_rev4a	CATCTGGTCCAGTAAATCGAATAATYGATCTTGACGCTGTTCAACACGTTTAAGKTSCTCATTACGTTGAGACAAAGGCTGTGATGGCGC
DBL2_L1	3ONJ_rev4b	CATCTGGTCCAGTAAATCGAATAATYGATCTTGCTBCTGTTCAACACGTTTAAGKTSCTCATTACGTTGAGACAAAGGCTGTGATGGCGC
DBL2_L1	3ONJ_rev4c	CATCTGGTCCAGTAAATCGAATAATYGATCTTGACGCTGTTCAACCTBTTTAAGKTSCTCATTACGTTGAGACAAAGGCTGTGATGGCGC
DBL2_L1	3ONJ_rev4d	CATCTGGTCCAGTAAATCGAATAATYGATCTTGCTBCTGTTCAACCTBTTTAAGKTSCTCATTACGTTGAGACAAAGGCTGTGATGGCGC
DBL2_L1	3ONJ_fw5	TTATTCGATTTACTGGACCAGATGGATGTGGAGGTTAATAACAGCATCGGGGACGCATCAGAACGCGCCACTTATAAAG
DBL2_L1	3ONJ_rev6	GCTTGATGTCGGACTGGATCGTTTTTTTCCACTCGCGTAACTTTGCTTTATAAGTGGCGCGTTCT
DBL2_L1	3ONJ_fw7	CCAGTCCGACATCAAGCGCCCGCTTCAGAGTTTGGTTGATAGTGGCGATGGATCCCAGGAACTGACAA
DBL2_L1	3ONJ_rev8	GAGTACGGCGTCGATTCTAAAGTTGGTGAGGGGATTTGCTCGCATATAGTTGTCAGTTCCTGGGATCC

Supplementary Table 7: Target protein sequences.

Protein target:	Sequence:	Notes:
PD-1 (Uniprot #Q15116)	LDSPDRPWNPPTFSPALLVVTEG<br>DNATFTCSFSDTSESFVLNWYRM<br>SPSDQTDKLAAFPEDRSQPGQDS<br>RFRVTQLPNGRDFHMSVVRARR<br>NDSGTYLCGAISLAPKAQIKESLR<br>AELRVTERRAEVPTAHPSPSPRPA<br>GQFQ	Mutated glycosylation site (N->D) and mutated free cysteines (C->S) underlined
CTLA4 (Uniprot #P16410)	KAMHVAQPAVVLASSRGIASFVC<br>EYASPGKATEVRVTVLRQADSQV<br>TEVCAATYMMGNELTFLDDSICT<br>GTSSGNQVNLTIQGLRAMDTGLY<br>ICKVELMYPPPYYLGIGDGTQIYVI<br>DPEPCPDSD	Mutated glycosylation site underlined (N->D)
RBD WT (Uniprot #P0DTC2)	RVQPTESIVRFPNITNLCPFGEVF<br>NATRFASVYAWNRKRISNCVADY<br>SVLYNSASFSTFKCYGVSPTKLNDL<br>CFTNVYADSFVIRGDEVRQIAPG<br>QTGKIADYNYKLPDDFTGCVIAW<br>NSNNLDSKVGGNYNYLYRLFRKS<br>NLKPFERDISTEIYQAGSTPCNGV<br>EGFNCYFPLQSYGFQPTNGVGYQ<br>PYRVVVLSFELLHAPATVCGPKKS<br>TNLVKNKCVNFNFNGLTGTGVLT<br>ESNKKFLPFQQFGRDIADTTDAV<br>RDPQTLEILDITPCS	
PD-L1 (Uniprot #Q9NZQ7)	SFTVTVPKDLYVVEYGSNMTIECK<br>FPVEKQLDLAALIVYWEMEDKNII<br>QFVHGEEDLKVQHSSYRQRARLL<br>KDQLSLGNAALQITDVKLQDAGV<br>YRCMISYGGADYKRITVKVNAPY<br>NKINQRILVVDPVTSEHELTCQAE<br>GYPKAEVIWTSSDHQVLSGKTTT<br>TNSKREEKLFNVTSTLRINTTTNEI<br>FYCTFRRLDPEENHTAELVIPELPL<br>AHPPNERTD	
