# Supplementary material for: De novo design of protein interactions with learned surface fingerprints
Source: Nature. 2023 Apr 26;617(7959):176–84. doi: 10.1038/s41586-023-05993-x (PMC10131520; doi:10.1038/s41586-023-05993-x)
Supplement: Supplementary file 2 — Reporting Summary [file 41586_2023_5993_MOESM2_ESM.pdf]

nature portfolio

Corresponding author(s): Bruno Correia

Last updated by author(s): 14/02/23

Reporting Summary

Nature Portfolio wishes to improve the reproducibility of the work that we publish. This form provides structure for consistency and transparency in reporting. For further information on Nature Portfolio policies, see our Editorial Policies and the Editorial Policy Checklist.

Statistics

For all statistical analyses, confirm that the following items are present in the figure legend, table legend, main text, or Methods section.

n/a	Confirmed	
☐	☒	The exact sample size (*n*) for each experimental group/condition, given as a discrete number and unit of measurement
☐	☒	A statement on whether measurements were taken from distinct samples or whether the same sample was measured repeatedly
☒	☐	The statistical test(s) used AND whether they are one- or two-sided<br>*Only common tests should be described solely by name; describe more complex techniques in the Methods section.*
☒	☐	A description of all covariates tested
☒	☐	A description of any assumptions or corrections, such as tests of normality and adjustment for multiple comparisons
☐	☒	A full description of the statistical parameters including central tendency (e.g. means) or other basic estimates (e.g. regression coefficient) AND variation (e.g. standard deviation) or associated estimates of uncertainty (e.g. confidence intervals)
☒	☐	For null hypothesis testing, the test statistic (e.g. *F*, *t*, *r*) with confidence intervals, effect sizes, degrees of freedom and *P* value noted<br>*Give P values as exact values whenever suitable.*
☒	☐	For Bayesian analysis, information on the choice of priors and Markov chain Monte Carlo settings
☒	☐	For hierarchical and complex designs, identification of the appropriate level for tests and full reporting of outcomes
☒	☐	Estimates of effect sizes (e.g. Cohen's *d*, Pearson's *r*), indicating how they were calculated

Our web collection on statistics for biologists contains articles on many of the points above.

Software and code

Policy information about availability of computer code

Data collection	XDS v Jan 10, 2022 (BUILT=20220220) package for processing of crystallographic data; Automation program EPU (ThermoFischer Sci., v2.12.1) for cryoEM data collection; Biacore 8K control software (Cytiva, v4.0.8.19879) for measuring surface plasmon resonance; LE-SH800SZFCPL Cell Sorter software (Sony, v2.1.5) for sorting and collecting FACS data; Kaluza for Galios (Beckman Coulter, v1.1.20388.18228) for collecting flowcytometry data; Chromeleon (ThermoFischer Sci, v7.2.10) and Astra (Wyatt Tech., v8.0.2.5) for performing and collecting data of SEC-MALS; GatorOne (Gator Bio, v.7.3.0728) for biolayer interferometry; Python (v3.6.5) as well as TensorFlow (v1.12) and PyMESH (v0.2.1) to run our MaSIF-seed pipeline; Custom scripts to collect protein modeling data are available on Github (https://github.com/LPDI-EPFL/masif_seed)
Data analysis	Coot (v0.9.5) for structure building; Prism (GraphPad, v9) for graphs generation; FlowJo (BD Bioscience, v10.8.1) for flowcytometry analysis; PyMol (Schrödinger, v2.0) and ChimeraX (UCSF, v1.3) for protein vizualization and structural graphic generation; MolProbity (v4.5.1) for structure evaluation; Phaser MR and Phenix.refine in Phenix (v1.19.2-4158 and v1.20.1-4487) for crystal structure determination and refinement; cryoSPARC (v3.3.1) for cryoEM structure determination, Biacore Insight Evaluation Software (Cytiva, v4.0.8.19879) for evaluating surface plasmon resonance measurements, Rosetta modelling suite (v3.13) for protein design and analysis; Custom scripts to analyze protein modeling data are available on Github (https://github.com/LPDI-EPFL/masif_seed)

For manuscripts utilizing custom algorithms or software that are central to the research but not yet described in published literature, software must be made available to editors and reviewers. We strongly encourage code deposition in a community repository (e.g. GitHub). See the Nature Portfolio guidelines for submitting code & software for further information.

Data

Policy information about availability of data

All manuscripts must include a data availability statement. This statement should provide the following information, where applicable:

- Accession codes, unique identifiers, or web links for publicly available datasets
- A description of any restrictions on data availability
- For clinical datasets or third party data, please ensure that the statement adheres to our policy

Cryo-EM maps were deposited in the Electron Microscopy Data Bank under the access codes of EMD-14947 (spikeD614G-binder full and spikeD614G-binder local maps), EMD-14922 (spikeOmicron-binder full), and EMD-14930 (spikeOmicron-binder local). Atomic models were deposited in Protein Data Bank under the access codes of PDB-7ZSS (spikeD614G-binder), PDB-7ZRV (spikeOmicron-binder full) and PDB-7ZSD (spikeOmicron-binder local). Crystal structures have been deposited in the Protein Data Bank under accession codes 7XYQ (DBL1_03/PD-L1 complex) and 7XAD (DBL2_02/PD-L1 complex). The PDBbind database (2018 released), PRISM database, ZDock benchmark and SabDab database are available with the following links respectively: http://pdbbind.org.cn/index.php, http://cosbi.ku.edu.tr/prism, https://zlab.umassmed.edu/benchmark/ and http://opig.stats.ox.ac.uk/webapps/sabdab. MaSIF-seed and the Rosetta design scripts are available at https://github.com/LPDI-EPFL/masif_seed. The scaffold database used for grafting the seeds provided by MaSIF-seed is available at https://zenodo.org/record/7643697#.Y-z533ZKhaQ

Human research participants

Policy information about studies involving human research participants and Sex and Gender in Research.

Reporting on sex and gender	N/A
Population characteristics	N/A
Recruitment	N/A
Ethics oversight	N/A

Note that full information on the approval of the study protocol must also be provided in the manuscript.

Field-specific reporting

Please select the one below that is the best fit for your research. If you are not sure, read the appropriate sections before making your selection.

☒ Life sciences ☐ Behavioural & social sciences ☐ Ecological, evolutionary & environmental sciences

For a reference copy of the document with all sections, see nature.com/documents/nr-reporting-summary-flat.pdf

Life sciences study design

All studies must disclose on these points even when the disclosure is negative.

Sample size	For the protein designs, 16 individual designs were tested for PD-L1, 20 individual designs for SARS-CoV2-RBD and 1500-2000 designs for the one-shot design approach targeting PD-L1, PD-1 and CTLA-4
Data exclusions	There is no data exclusion in this study.
Replication	Each binding candidate detected by yeast display and/or deep-sequencing showed reproducible results and were also tested with negative controls (unrelated protein ligand or unlabeled yeast) and point mutants. Result reproducibility was also confirmed with alternative methods (e.g. SPR)
Randomization	Oligos encoding the one-shot designs were pooled together (one oligopool per target) and then sorted by yeast display
Blinding	Researchers were not blinded as this does not apply to our study (no bias expected from cell sorting)

Reporting for specific materials, systems and methods

We require information from authors about some types of materials, experimental systems and methods used in many studies. Here, indicate whether each material, system or method listed is relevant to your study. If you are not sure if a list item applies to your research, read the appropriate section before selecting a response.

Materials & experimental systems

n/a	Involved in the study
☐	☒ Antibodies
☐	☒ Eukaryotic cell lines
☒	☐ Palaeontology and archaeology
☒	☐ Animals and other organisms
☒	☐ Clinical data
☒	☐ Dual use research of concern

Methods

n/a	Involved in the study
☒	☐ ChIP-seq
☐	☒ Flow cytometry
☒	☐ MRI-based neuroimaging

Antibodies

Antibodies used

1) Anti-HA, FITC - Ref : A190-138F - Manufacturer : Bethyl - Clone : Unknown
2) Anti-V5 mouse - Ref : MA5-15253 - Manufacturer : Invitrogen - Cline : E10/V4RR
3) Anti-mouse, FITC - Ref : F0257 - Manufacturer : Sigma - Clone : Polyclonal
4) Anti-His, PE - Ref : 130-120-787 - Manufacturer : Miltenyi Biotec - Clone : GG11-8F3.5.1
5) Anti-Myc, FITC - Ref : SAB4700448 - Manufacturer : Sigma - Clone : 9E10
6) Anti-human IgG, PE - Ref : 12-4998-82 - Manufacturer : Invitrogen - Clone : Polyclonal
7) Anti-Human IgG, R-PE - Ref : 109-117-008 - Manufacturer : Jackson ImmunoResearch - Clone : Polyclonal

Validation

For the commercially available antibodies, no validation reports were provided other than publications citing the products or examples on manufacturer's webpage :
1) 10.21037/atm.2020.03.74
2) 10.1038/s41467-021-22969-5
3) 10.1101/gad.1575307
4) https://www.miltenyibiotec.com/US-en/products/his-antibody-gg11-8f3-5-1.html#pe:100-tests-in-200-ul
5) 10.1039/c8nr03970d
6) https://www.thermofisher.com/antibody/product/Goat-anti-Human-IgG-Fc-Secondary-Antibody-Polyclonal/12-4998-82
7) 10.1038/s41467-020-19231-9

Eukaryotic cell lines

Policy information about cell lines and Sex and Gender in Research

Cell line source(s)

Karpas-299 purchased from Sigma (Ref: 06072604-1VL) with ECACC (European Collection of Authenticated Cell Cultures) approval
Expi293 purchased from ThermoFischer Sci. (Ref: A14635)

Authentication

Authenticated by the provider and no additional authentication has been done

Mycoplasma contamination

Karpas-299 : Cells were tested negative for mycoplasma contamination by the manufacturer (PCR) and no additional test was performed as the cells were directly used for a single experiment after purchase.
Expi299: Cells were tested negative for mycoplasma contamination by the manufacturer (qPCR) and no additional test was performed as the cells were used for protein expression.

Commonly misidentified lines
(See ICLAC register)

No commonly misidentified lines were used in this study

Flow Cytometry

Plots

Confirm that:

☒ The axis labels state the marker and fluorochrome used (e.g. CD4-FITC).

☒ The axis scales are clearly visible. Include numbers along axes only for bottom left plot of group (a 'group' is an analysis of identical markers).

☒ All plots are contour plots with outliers or pseudocolor plots.

☒ A numerical value for number of cells or percentage (with statistics) is provided.

Methodology

Sample preparation

Yeasts cells (EBY-100) were labeled with the protein target and then with an anti-Myc (FITC), anti-V5 (FITC) or anti-HA (FITC) for the display signal and an anti-Fc (PE) or anti-His (PE) for binding to the protein target. Cells were washed with PBS supplemented with 0.1% BSA. See methods for more details

Instrument

Sony SH800 (Sorting) and Beckman Coulter Gallios (Analysis)

Software	Sony LE-SH800SZFCPL Cell Sorter software (v2.1.5), Kaluza for Gallios (v1.1.20388.18228) and FlowJo (v10.8.1)
Cell population abundance	Transformed yeasts underwent several round of sorts for target binding and amplifications in culture (2 to 3 cycles) before being sequenced for the isolation of single binding clones. Single clone candidates (obtained from a single colony on plate or re-transformation of naive yeasts with pure DNA) were then individually tested with controls.
Gating strategy	Yeast cells without target labeling served as a negative control and yeasts showing binding signal above this negative threshold were collected (See Extended Data Fig. 3a-b for an example).

☒ Tick this box to confirm that a figure exemplifying the gating strategy is provided in the Supplementary Information.
